# Diversity of Plant-Based Food Consumption: A Systematic Scoping Review on Measurement Tools and Associated Health Outcomes

**DOI:** 10.1093/nutrit/nuaf040

**Published:** 2025-04-29

**Authors:** Alice C Creedon, Vienna Hubbard, Rachel Gibson, Eirini Dimidi

**Affiliations:** Department of Nutritional Sciences, King’s College London, London, SE1 9NH, United Kingdom; Department of Nutritional Sciences, King’s College London, London, SE1 9NH, United Kingdom; Department of Nutritional Sciences, King’s College London, London, SE1 9NH, United Kingdom; Department of Nutritional Sciences, King’s College London, London, SE1 9NH, United Kingdom

**Keywords:** diversity, variety, plant-based foods, plant-based diet, diet quality

## Abstract

A scoping review of the literature was conducted to identify studies investigating plant-based food diversity and human health outcomes. Objectives were to (a) explore definition(s) of plant-based foods used, (b) identify assessment tools used to measure plant-based food consumption, (c) characterize the assessment tools and methodology used to measure the diversity of plant-based food consumption, and (d) identify the health outcomes that have been investigated in relation to the diversity of plant-based food consumption, and explore the findings. Consumption of diverse plant-based foods provides a rich source of nutrients and nonnutrient bioactives that are often reported to improve health outcomes. Despite this, there are no standard definitions of plant-based food diversity, there is no consensus on methods of measurement, and there is limited understanding of its associated health benefits. Eligible studies were those investigating the relationship between plant-based food diversity and any health-related or lifestyle outcome, by any study design, in high income countries only. Studies were identified by systematic searches of 2 electronic databases and manual searches of reference lists. No restrictions were applied for language or year of publication. Forty-three studies were eligible for inclusion in this review. The majority of the studies were observational in design (38/43; 88%) and included fruits and vegetables only in their definitions of plant-based food diversity (31/43, 72%). Methods of measurement of both plant-based food intake and diversity varied greatly between studies, with only 3 (7%) articles reporting the use of a dedicated tool for assessment of plant-based food diversity in their population of interest. Health outcomes assessed included dietary intake and behavior, cardiometabolic risk factors, socioeconomic determinants of health, and cancer risk. There is a need for a robust definition and standardized assessment tools for plant-based food diversity. Observational studies have found associations between plant-based food diversity and certain health outcomes that warrant investigation in future randomized controlled trials.

## INTRODUCTION

Plant-based foods encompass all food products derived from plant sources, such as fruits, vegetables, grains, legumes, herbs and spices, nuts and seeds, plant-based fats and oils (eg, olive oil) and plant-based beverages (eg, tea and coffee). Consumption of plant-based foods has soared in recent years, as reflected by a 60% increase in sales from 2017 to 2020.^1^ There is evidence indicating benefits of diets rich in healthful plant-based foods, including association with lower mortality, and reduced cancer and cardiovascular disease risk.^2^ A dose–response health benefit has also been identified for certain individual plant-based food groups, such as fruits and vegetables, with a 200 g/d increment increase resulting in a 15% reduction in relative risk of all-cause mortality.^3^

In addition to quantity of plant-based food consumption, interest in diversity of plant-based food consumption has increased in recent years. Although no formal definition exists, plant-based food diversity refers to the variety of different plant-based food items or food groups consumed over a given time period. Preliminary evidence suggests plant-based food diversity may be associated with health benefits, such as a higher gut microbiota α-diversity and a lower incidence of type 2 diabetes mellitus.^4^^,^^5^ Plant-based foods contain fiber, vitamins, minerals, and bio-active phytochemicals, in varying forms and quantities.^6^ The benefits of diverse plant-based food consumption may relate to the notion that intake of different plant-based foods may ensure adequate intake of essential nutrients, and result in a synergistic effect between micronutrients, which can enhance absorption or have cumulative effects beyond the specific micronutrient alone.^7^^,^^8^ Indeed, national and international dietary guidelines recommend the consumption of a “variety” of fruits and vegetables.^9^^,^^10^ However, these recommendations are limited to fruits and vegetables only, and do not expand to other plant-based foods with known health benefits, such as nuts and seeds. The reason for this is likely that, although evidence exists on the health benefits of such foods, there is limited research on the impact of consuming a diverse range.

The limited evidence available on plant-based food diversity and the associated health effects may be attributed to several factors. First, there is a lack of a standardized definition of plant-based foods, as well as of diversity of plant-based food consumption, and characterization of the specific foods and food groups included/excluded in this definition. Second, there is a lack of a standardized and validated methodology of measuring plant-based food intake and diversity, primarily in terms of the assessment tools and analyses used. To address these limitations, this study aimed to identify and characterize the methods used to measure plant-based food diversity, and to identify the health outcomes associated with plant-based food diversity, via a scoping review of the literature. This was undertaken with the following objectives: (a) to explore the definition(s) of plant-based foods, (b) to identify the assessment tools used to measure plant-based food consumption, (c) to characterize the assessment tools and methodology used to measure diversity of plant-based food consumption, and (d) to identify the health outcomes that have been investigated in relation to the diversity of plant-based food consumption and explore the findings.

## METHODS

The protocol for this systematic scoping review was developed by 3 of the researchers (E.D., R.G., V.H.) prior to conducting the systematic searches, and is available from the corresponding author upon request. The study is reported in line with the guidelines for Preferred Reporting Items for Systematic Reviews and Meta-Analyses Extension for Scoping Reviews (PRISMA-ScR).^11–13^

### Eligibility Criteria

The eligibility criteria were developed using a Patient, Exposure, Comparators, Outcome, and Study Design (PECOS) approach and are outlined in Table 1.^14^ Briefly, the inclusion criteria were any observational or intervention studies for which the measurement of plant-based food diversity was reported, as well as the effect of plant-based food diversity on health-related outcomes, in any age group. Health-related outcomes included clinical outcomes, as well as indicators of health status (such as health claims) and determinants of health (such as dietary intake and socioeconomic status). Only studies conducted in high-income countries, as defined by the World Bank,^14^ were included. Since food systems differ significantly in relation to countries’ socioeconomic status, focusing on high-income countries ensured as homogeneous a sample population as possible, and maximized the comparability of the study findings.

**Table 1.** The PECOS Criteria for Inclusion of Studies

Parameter	Inclusion criterion	Exclusion criterion	Data extracted
Population	People of any age living in high-income countries^14^		Age, sex, country, ethnicity
Exposure	Plant-based food diversity assessment		Definition of plant-based foods, definition of plant-based food diversity, characterization of plant-based food diversity and quantity, assessment tools for plant-based food diversity measurement
Comparators	NR		NR
Outcomes	Any health-related or lifestyle outcomes		Health/disease/lifestyle outcomes assessed and the association with / impact of plant-based food diversity intake
Study design	Observational or interventional studies. No restrictions were applied to language or year of publication	Animal studies, Reviews, Expert opinions, Comments, Letters to the editor, and Conference reports were excluded	Study design, sample size, language, and year of publication

Abbreviation: NR, not relevant.

### Search Strategy

Studies were identified through a systematic search of electronic databases, and hand-searching of reference lists of eligible studies and relevant review papers. Two electronic databases were searched for eligible studies: Web of Science (1900 to October 2022; Web of Knowledge portal) and Medline (1946 to October 2022; OvidSP). The final search date was October 28, 2022. Combinations of terms related to plant-based foods, dietary diversity, and dietary quality were used as medical subject headings and free-text terms. The detailed search strategies are presented in Table S1. No restrictions were applied to language or publication date.

### Selection Process

References were imported into a reference manager for assessment of eligibility (EndNote 20; Thomson Reuters). Following automatic and manual removal of duplicates, a reviewer (V.H.) screened the titles and abstracts and then the full-text articles against the predefined inclusion and exclusion criteria. Uncertainties about eligibility were resolved by discussion among the research team (E.D., R.G., V.H., A.C.C.).

### Data Collection Process

All relevant information from the eligible studies was extracted into a standardized form (V.H., A.C.C.). The data extracted included population characteristics and study design, method used to define plant-based foods, dietary assessment tool, diversity assessment tool, and health outcome(s) measured. Where a full paper or abstract provided insufficient data, the authors were contacted to provide additional information.

### Data Synthesis

A qualitative and descriptive approach was adopted to review the available evidence regarding the assessment of plant-based food diversity and its associated health benefits. Studies were characterized in terms of study design, population demographics, and country of origin. The assessment tools and methodology of plant-based food quantity and/or diversity measurement, as well as the associated health outcomes, were characterized for each study. Qualitative synthesis to map the literature and outline approaches in the data was followed to identify assessment tools used and health outcomes assessed.

## RESULTS

**Figure 1.**
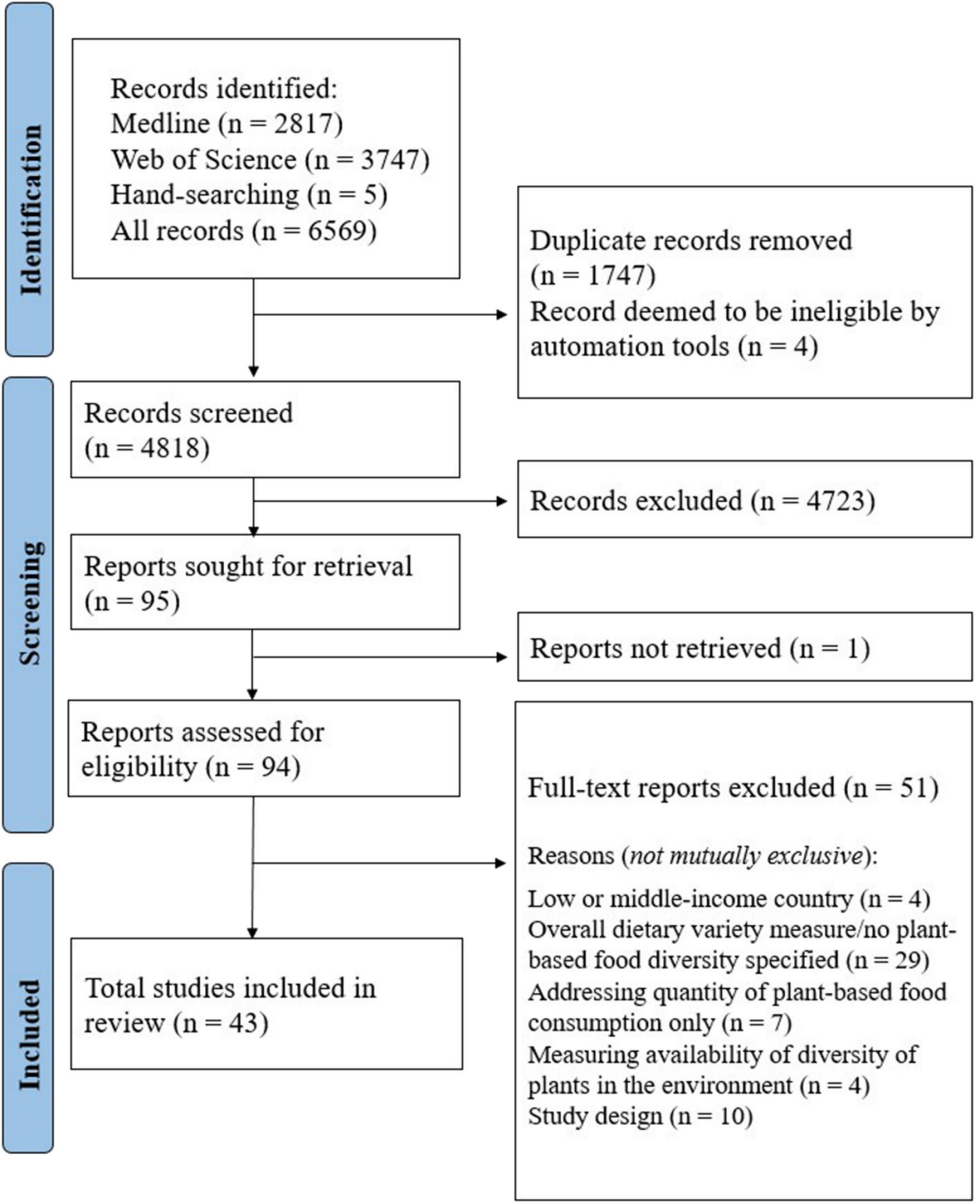
PRISMA Flow Diagram of Studies Included in a Scoping Review

A total of 6569 records (including duplicates) were identified in the electronic and hand searches. Following the screening of titles and abstracts, 95 of the records were deemed to be potentially eligible for inclusion, of which 51 were excluded and 1 could not be retrieved. In total, 43 records fulfilled the criteria for inclusion in the review, published from 1994 to 2022. The number of records identified and the reasons for exclusion at each stage are presented in Figure 1. The characteristics of eligible studies are presented in Table S2. A total of 17/43 (40%) records were cross-sectional studies,^4^^,^^15–30^ 14/43 (33%) were prospective cohort studies,^5^^,^^31–43^ 7/43 (16%) were case–control studies,^44–50^ 2/43 (5%) involved secondary analyses of randomized controlled trials (RCTs),^51^^,^^52^ 2/43 (5%) were nonrandomized controlled trials,^53^^,^^54^ and 1/43 (2%) was a case study.^55^ Sample sizes ranged from 63 to 452 269 participants, and studies were conducted in the USA,^4^^,^^18^^,^^20^^,^^21^^,^^27^^,^^29–31^^,^^37^^,^^42^^,^^49^^,^^50^ Europe,^5^^,^^16^^,^^17^^,^^19^^,^^23^^,^^25^^,^^26^^,^^28^^,^^32–35^^,^^38–41^^,^^43–48^^,^^53^ Australia,^15^^,^^22^^,^^24^^,^^36^^,^^51^^,^^52^^,^^55^ and New Zealand.^54^ Twelve authors were contacted to provide additional information about 13 articles,^4^^,^^5^^,^^15–18^^,^^31–36^^,^^44^ and 2 of them responded.^4^^,^^36^

### Definition of Plant-Based Foods

Definitions of plant-based foods varied across studies. Thirty-two (74%) studies counted fruits and vegetables only in their definitions of plant-based foods.^16^^,^^18–24^^,^^26–30^^,^^32–43^^,^^45^^,^^48^^,^^51–55^ Of the remaining studies, 5 (12%) counted fruits, vegetables, and grains,^5^^,^^31^^,^^47^^,^^49^^,^^50^ while 3 (7%) studies specified fruit, vegetables, and cereals, when defining plant-based foods.^25^^,^^44^^,^^46^ One study included vegetables only.^15^ One study included plant-based foods from 7 groups: Grains, legumes, nuts and seeds, fruits, vegetables, potatoes, and other vegetables.^17^ Another did not provide details of their definition of plant-based foods beyond stating that it included “the number of unique plant species consumed per week”.^4^ It was unclear in all studies whether definitions included herbs and spices, plant-based fats and oils, or plant-based beverages.

Studies assessed diversity in a variety of ways (Table 2), for example, at the item level (eg, broccoli, lettuce, apple), at the subgroup level (eg, cruciferous vegetables, citrus fruits), and/or at the group level (eg, vegetables, fruits). Of studies that assessed plant-based food items in their definition of diversity, the total number of items ranged from 21 to 223.^16^^,^^19^^,^^21^^,^^22^^,^^24^^,^^27^^,^^32–34^^,^^36–41^^,^^43^^,^^46^^,^^47^^,^^50^^,^^53^ Of studies that assessed plant-based food subgroups, the total number of fruit subgroups (eg, citrus, noncitrus) ranged from 2 to 30, and the total number of vegetable subgroups (eg, green leafy, cruciferous) ranged from 1 to 36.^5^^,^^19^^,^^21^^,^^23^^,^^25^^,^^27^^,^^29^^,^^30^^,^^33–35^^,^^40^^,^^41^^,^^43^^,^^48^^,^^51^^,^^52^ One study assessed cereals as 3 subgroups (potatoes, refined cereals, and wholegrain cereals),^25^ and another assessed grains as 2 subgroups (wholegrains and non-wholegrains).^5^ Several studies assessed a mixture of plant-based food items and subgroups when defining diversity.^27^^,^^43^^,^^45^ One study assessed plant-based diversity at the level of 7 subgroups contributing to plant-based protein intake (grains, legumes, nuts and seeds, fruits, vegetables, potatoes, and other vegetables).^17^ Two studies assessed diversity at the level of 1 plant-based food group (vegetables).^15^^,^^18^ Four studies found diversity at the level of the plant-based food group but did not provide further details of food items or subgroups included in their definitions.^20^^,^^31^^,^^42^^,^^44^ Three articles reported assessing plant-based food items; 2 of these reported diversity at the level of the plant-based food group (fruits, vegetables),^54^^,^^55^ but in none of the articles were further details on food items/subgroups included in the assessment reported.^26^ One study assessed plant-based food diversity as the number of different “plants” consumed per week, but did not provide further detail on items or subgroups included.^4^

**Table 2.** Measurement Tools for Assessment of Diet and Plant-Based Diversity

Study	Dietary assessment tool	Number of food items included in assessment tool	Time period for dietary assessment	Method of assessment of plant-based food diversity	Number of plant-based food items/subgroups/groups included in assessment of diversity	Quantity cut-off for inclusion in diversity score	Time period for diversity assessment
Aljadani et al (2013)^36^	FFQ (DQESv2)^a^	74	12 months	Fruit and Vegetable Index (FAVI)	Fruits: 13 items Vegetables: 24 items Total items: 37 items Total groups: 2	No minimum quantity	Per month
Almeida de Souza et al (2018)^19^	FFQ^a^	91	12 months	Sum of unique plant-based items consumed	Fruits: 12 subgroups (19 items) Vegetables: 15 subgroups (22 items) Total items: 41 Total subgroups: 27 Total groups: 2	No minimum quantity	Per month
Baldwin et al (2021)^24^	FFQ (DQESv2)^a^	74	12 months	Fruit and Vegetable Variety Index (FAVVA)	Fruits: 11 items Vegetables: 24 items Total items: 35 Total groups: 2	Portion size (e.g. 1 banana)	Per month
Bernstein et al (2002)^20^	Weighed food diary^a^	N/A	3 d	Sum of unique plant-based items consumed	Fruits and Vegetables Further information NR Total groups: 2	No minimum quantity	3 d
Bhupathiraju et al (2013)^37^	FFQ^a^	126	7 d	Sum of unique plant-based items consumed	Fruits: 5 items Vegetables: 21 items Total items: 26 Total groups: 2	No minimum quantity	Weekly
Buchner et al (2010)^33^	Country-dependent. Quantitative dietary questionnaire; semi-quantitative FFQ; 7-d food diary^a^	88-2443 (assessment method-dependent)	12 months; 14 d; 7 d	Sum of unique plant-based items/subgroups consumed	Fruits: 3 subgroups (21 items) Vegetables: 8 subgroups (38 items) Total items: 59 Total subgroups: 11 Total groups: 2	No minimum quantity	2 wks
Buchner et al (2011)^34^	Country-dependent. Quantitative dietary questionnaire; semi-quantitative FFQ; 7-d or 14-d food diary^a^	88-2443 (assessment method-dependent)	12 months; 14 d; 7 d	Sum of unique plant-based items/subgroups consumed	Fruits: 3 subgroups (21 items) Vegetables: 8 subgroups (38 items) Total items: 59 Total subgroups: 11 Total groups: 2	No minimum quantity	2 wks
Byrne et al (2018)^52^	24-h diet recall, 2-d food diary^a^	N/A	3 d	Sum of unique plant-based subgroups consumed	Fruits: 30 subgroups Vegetables: 36 subgroups Total subgroups: 66 Total groups: 2	No minimum quantity	3 d
Cano-Ibanez et al (2019)^25^	FFQ^a^	143	12 months	Sum of unique plant-based subgroups consumed	Fruits: 3 subgroups Vegetables: 4 subgroups Cereals: 3 subgroups Total subgroups: 10 Total groups: 3	Half of portion size during 1 d	Per month
Conklin et al (2014)^16^	FFQ^a^	NR	1 month	Sum of unique plant-based items consumed	Fruits: 11 items Vegetables: 26 items Total items: 37 Total groups: 2	No minimum quantity	Per month
Conklin et al (2016)^5^	FFQ^a^	130	12 months	Sum of unique plant-based subgroups consumed	Fruits: 4 subgroups Vegetables: 4 subgroups Grains: 2 subgroups Total subgroups: 10 Total groups: 3	No minimum quantity	Per month
Cooper et al (2012)^38^	Food diary^a^	N/A	7 d	Sum of unique plant-based items consumed	Fruits: 58 items Vegetables: 59 items Total items: 117 Total groups: 2	No minimum quantity	Weekly
De Castro-Mendez et al (2021)^23^	24-h dietary recall^a^	N/A	24 h	Sum of unique plant-based subgroups consumed	Fruits: 2 subgroups Vegetables: 4 subgroups Total subgroups: 6 Total groups: 2	≥15 g	Daily
Ellis et al (2018)^21^	National Cancer Institute Fruit & Vegetable All-Day screener^a^	107	1 month	Sum of unique plant-based items consumed, expressed as a proportion of foods consumed from each fruit/vegetable subgroup	Fruit: 2 subgroups (22 items) Vegetable: 8 subgroups (33 items) Total items: 55 Total subgroups: 10 Total groups: 2	No minimum quantity	Monthly
Estaquio et al (2008)^40^	24-h dietary recall^a^	N/A	2 y	Sum of unique plant-based items consumed, expressed as a proportion of foods consumed from each fruit/vegetable subgroup	Fruits: 9 subgroups (39 items) Vegetables: 10 subgroups (50 items) Total items: 89 Total subgroups: 19 Total groups: 2	Unclear	Unclear
Fernandez et al (1996)^47^	Structured questionnaire	29	Weekly	Sum of unique plant-based items consumed	Fruit: 3 items Vegetables: 8 items Grains: 4 items Total items: 15 Total groups: 3	No minimum quantity	Weekly
Fernandez et al (2016)^31^	Harvard Service FFQ^a^	84	1 month	Sum of unique plant-based items consumed, adjusted for evenness of food subgroup distribution (Berry Index)	Fruits, Vegetables, Grains Further information NR Total groups: 3	Standard portion size	Weekly
Garavello et al (2008)^48^	FFQ^a^	78	2 y	Sum of unique plant-based items consumed	Fruits: 4 subgroups Vegetables: 4 subgroups Further information NR Total subgroups: 8	No minimum quantity	Weekly
Garavello et al (2009)^44^	FFQ^a^	78	2 y	Sum of unique plant-based items consumed	Fruits, Vegetables, Cereals Further information NR Total groups: 3	No minimum quantity	Weekly
Hazley et al (2022)^26^	Semi-weighed food diary^a^	N/A	4 d	Sum of unique plant-based items consumed	NR	No minimum quantity	4 d
Hoy et al (2020)^18^	24-h dietary recall^a^	N/A	24 h	Sum of unique plant-based items consumed; each fruit and vegetable counted once; a mixed dish counted as 1 fruit/vegetable	Fruits and Vegetables Further information NR Total groups: 2	≥0.1 cup equivalent for single items ≥0.2 cup equivalent for items in mixed dishes	Daily
Hurley et al (2010)^27^	24-h dietary recall^a^	N/A	24 h	Sum of unique plant-based food items consumed	Fruit: 4 subgroups (8 items) Vegetables: 5 subgroups (13 items) Total items: 21 Total subgroups: 9 Total groups: 2	No minimum quantity	Daily
Jamison et al (2003)^55^	Dietary questionnaire	NR	24 h	Sum of unique plant-based items consumed	Fruits, Vegetables Further information NR Total groups: 2	No minimum quantity	Daily
Jansen et al (2004)^32^	FFQ	34	1 month	Sum of unique plant-based items consumed	Fruits: 7 items Vegetables: 27 items Total items: 34 Total groups: 2	No minimum quantity	Per month
Jeurnick et al (2012)^43^	Country-dependent. Quantitative dietary questionnaire; semi-quantitative and nonquantitative FFQ; 14-d food diary^a^	N/A	12 months; 14 d	Sum of unique plant-based items/subgroups consumed	Fruit: 14 items Vegetables: 8 subgroups (26 items) Total items: 40 Total subgroups: 8 Total groups: 2	No minimum quantity	2 wks
La Vecchia et al (1997)^45^	FFQ	29	1 y	Sum of unique plant-based items/subgroups consumed	Fruit: 2 items, 1 subgroup Vegetables: 5 items, 3 subgroups Total items: 7 Total subgroups: 4 Total groups: 2	No minimum quantity	Weekly
Leenders et al (2015)^41^	Dietary questionnaire, 7-day food diary^a^	N/A	12 months; 7 d	Sum of unique plant-based items/subgroups consumed	Fruit: 5 subgroups (16 items) Vegetables: 8 subgroups (33 items) Total items: 49 Total subgroups: 13 Total groups: 2	No minimum quantity	2 wks
Leslie et al (2012)^22^	Cancer Council of Victoria’s DQESv3 FFQ^a^	137	12 months	Sum of unique plant-based items consumed. Consuming fruits and vegetables “never” or “less than once per month” was coded as 0; consuming fruits and vegetables 1-3 times/month was coded as 1	Fruits: 21 items Vegetables: 29 items Total items: 50 Total groups: 2	Standard portion size	Per month
Lopez Gonzalez et al (2021)^28^	FFQ^a^	143	Unclear	Sum of unique plant-based items consumed	Fruits: 10 items Vegetables: 11 items Total items: 21 Total groups: 2	No minimum quantity	Per month
Lucenteforte et al (2008)^46^	FFQ^a^	78	2 y	Sum of unique plant-based items consumed	Fruits: 12 items Vegetables: 13 items Cereals: 13 items Total items: 38 Total groups: 3	No minimum quantity	Weekly
Marshall et al (2022)^30^	Texas School Physical Activity and Nutrition Survey questionnaire^a^	NR	24 h	Sum of unique plant-based subgroups consumed	Fruit: 2 subgroups Vegetables: 5 subgroups Total subgroups: 7 Total groups: 2	No minimum quantity	Daily
McCann et al (1994)^50^	FFQ^a^	128	12 months	Sum of unique plant-based items consumed	Fruits: 20 items Vegetables: 38 items Grains: 13 items Total items: 71 Total groups: 3	No minimum quantity	Per month
McDonald et al (2018)^4^	FFQ^a^	156	3 months	Self-reported number of different plants consumed. Single question	NR	No minimum quantity	Per week
Morison et al (2018)^54^	Weighed food diary^a^	N/A	3 d	Sum of unique plant-based items consumed	Fruits and vegetables Further information NR Total groups: 2	No minimum quantity	3 d
Oude Griep et al (2012)^39^	FFQ^a^	178	12 months	Sum of unique plant-based items consumed. One point for items consumed ≥1 times per 2 wks	Fruits: 9 items Vegetables: 20 items Total items: 29 Total groups: 2	No minimum quantity	2 wks
Perry et al (2015)^51^	24-h dietary recall^a^, 2-d food diary^a^	N/A	3 d	Sum of unique plant-based subgroups consumed	Fruits: 30 subgroups Vegetables: 36 subgroups Total subgroups: 66 Total groups: 2	No minimum quantity	3 d
Radavelli-Bagatini et al (2022)^15^	Self-reported daily vegetable diversity	1 group	24 h	Self-reported daily vegetable diversity; single question	Total groups: 1	No minimum quantity	Daily
Ramsay et al (2017)^29^	24-h dietary recall^a^	N/A	24 h	Sum of unique plant-based subgroups consumed	Fruits: 3 subgroups Vegetables: 4 subgroups Total subgroups: 7 Total groups: 2	No minimum quantity	Daily
Rigal et al (2021)^53^	24-h dietary recall^a^	N/A	4 d	Sum of unique plant-based items consumed	Fruits: 38 items Vegetables: 44 items Total items: 82 Total subgroups: 2	No minimum quantity	4 d
Salome et al (2020)^17^	24-h dietary recall^a^	N/A	3 d	(a) Sum of unique plant-based items/subgroups consumed, and taking into account (b) evenness of distribution (Berry Index)	Fruit and vegetables contributing to plant-based protein intake: 7 subgroups Total groups: 2	Unclear	3 d
Skinner et al (2002)^42^	24-h dietary recall^a^, 2-d food diary^a^	N/A	3 d	Variety Index for Children (modified)	Fruits and Vegetables. Further information NR Total groups: 2	≥¼ of standard portion size	9 d
Slattery et al (1997)^49^	Diet history questionnaire	>800, data collection on consumed foods only	1 y	Sum of unique plant-based items consumed	Fruits: 48 items Vegetables: 70 items Whole grains: 39 items Refined grains: 66 items Total items: 223 Total subgroups: 2 Total groups: 3	No minimum quantity	Unclear
Venter et al (2020)^35^	Questionnaire	N/A	3 months	Sum of unique plant-based subgroups consumed	Fruits: 3 subgroups Vegetables: 1 group Total subgroups: 3 Total groups: 2	No minimum quantity	3 months

a Indicates measurement method stated as validated; † examples of plant-based food items include broccoli, lettuce, and cauliflower; examples of plant-based food subgroups include leafy vegetables, and citrus fruits; examples of plant-based food groups include fruits, vegetables, and nuts.

Abbreviations: DQESv2/3, Dietary Questionnaire for Epidemiological Studies Version 2/3; FFQ, food frequency questionnaire; N/A, not applicable; NR, not reported.

Plant-based foods commonly reported to have been excluded from definitions of plant-based diversity include potatoes, fruit juices, legumes, dried fruits, nuts and seeds, olives and smoothies (Table S3).

In 22/43 (51%) studies, foods included in definitions of plant-based diversity were based on national dietary guidelines, including those of Australia,^15^^,^^24^^,^^36^^,^^51^^,^^52^ the United States,^18^^,^^30^^,^^37^^,^^42^^,^^47^ the Netherlands,^32^^,^^39^ Spain,^25^^,^^28^ Ireland,^26^ and the United Kingdom,^5^ or international guidelines provided by the European Union^41^^,^^43^^,^^44^ and the World Health Organization.^38^ A total of 9/43 (21%) studies considered seasonality of food items/subgroups or groups when defining plant-based diversity (Table S3).^19^^,^^32–34^^,^^39–41^^,^^43^^,^^46^

### Measurement Tools for Plant-Based Food Consumption

Studies utilized a variety of dietary assessment tools for the measurement of plant-based food intake (Table 2). The majority of studies (21/43, 49%) used food frequency questionnaires (FFQs) to assess plant-based food intake; all articles reported the FFQ used to be a validated tool.^4^^,^^5^^,^^15^^,^^16^^,^^19^^,^^22^^,^^24^^,^^25^^,^^28^^,^^31–34^^,^^36^^,^^37^^,^^39^^,^^43^^,^^44^^,^^46^^,^^48^^,^^50^ Three of these studies used FFQs in conjunction with other dietary assessment tools.^33^^,^^34^^,^^43^ The number of specific plant-based food items ranged from 21 to 71. One article did not report information on the length of the FFQ or the mode of utilization.^16^

Ten (23%) studies used 24-hour dietary recall to measure plant-based food intake; 7 of these studies used the 24-hour recall as the only method,^17^^,^^18^^,^^23^^,^^27^^,^^29^^,^^40^^,^^53^ while the remain 3 studies used this method in conjunction with another.^42^^,^^51^^,^^52^ This method was most commonly administered on a single occasion^18^^,^^23^^,^^27^^,^^29^^,^^42^^,^^51^^,^^52^; however 1 study analyzed data from participants who completed a total of 6 24-hour recalls over a period of 2 years,^40^ another administered 24-hour recalls 3 times over a period of 3 weeks,^17^ and another administered 24-hour recalls 4 times over an unspecified period.^53^

Food diaries were used to assess plant-based food intake in 11 (26%) studies.^20^^,^^26^^,^^33^^,^^34^^,^^38^^,^^41–43^^,^^51^^,^^52^^,^^54^ Of these, 5 used the food diary as the sole method of dietary assessment,^20^^,^^26^^,^^38^^,^^41^^,^^54^ while 3 studies used it in combination with 24-hour recall,^42^^,^^51^^,^^52^ and the remaining 3 studies used the diary in combination with either semi-quantitative^33^^,^^34^ or nonquantitative FFQs,^34^^,^^43^ depending on the country of administration. Food diaries were completed for a mean duration of 6 days (range 2–14 days). Two articles reported the use of weighed food diaries,^20^^,^^54^ 1 article reported the use of semi-weighed food diaries,^26^ and 2 studies provided visual aids to assist participants in estimating portion sizes.^38^^,^^51^ The remaining 4 studies did not provide further details on instructions given to participants to assist their recording. Three articles reported asking participants to include at least 1 weekend day in their record ^42^^,^^51^^,^^54^; 2 studies specified recording of the participants’ diet over either consecutive^20^ or nonconsecutive days.^54^

Ten (23%) articles reported using a “dietary questionnaire” to assess intake of plant-based foods.^21^^,^^30^^,^^33–35^^,^^41^^,^^43^^,^^47^^,^^49^^,^^55^ Four of these studies used a dietary questionnaire in combination with another method when analyzing plant-based food intake.^33^^,^^34^^,^^41^^,^^43^ Four studies, all investigating diet in an international sample incorporating multiple countries, used a variety of self-administered quantitative dietary questionnaires that reflected local diets.^33^^,^^34^^,^^41^^,^^43^ Other questionnaires that were used included a study-specific questionnaire (Texas School Physical Activity and Nutrition Survey) validated prior to use against 24-hour recall,^30^ a Fruit and Vegetable screener developed by the National Cancer Institute,^21^ and an adaptation of a questionnaire validated for use in a previous study.^49^ Three studies provided no details of the questionnaire used.^35^^,^^47^^,^^55^

### Methods of Measurement of Plant-Based Food Diversity

A total of 26/44 (59%) studies included only plant-based foods in their assessment of diversity.^4^^,^^15^^,^^16^^,^^18–21^^,^^23^^,^^24^^,^^28–30^^,^^32–43^^,^^53^^,^^55^ The remaining 17/43 (40%) studies included additional non-plant-based foods to form a total dietary diversity score, and the articles subsequently reported diversity scores for plant-based foods separately.^5^^,^^17^^,^^22^^,^^25–27^^,^^31^^,^^44–52^^,^^54^

Methods of measurement of plant-based food diversity identified in this review are reported, along with their respective advantages and disadvantages, in Table S4. Of the 43 studies included in this review, 3 (7%) studies assessed plant-based food diversity using a dedicated index of plant-based diversity (Table 2).^24^^,^^36^^,^^42^ One study utilized a diversity index developed based on national dietary guidelines in the United States,^42^ which strongly correlated with nutrient adequacy assessed by interview^56^ and assessed diversity of fruit and vegetable consumption separately, taking into consideration frequency of consumption. A second study, conducted in Australia, developed a novel fruit and vegetable variety index (FAVI) based on foods commonly consumed in the Australian diet.^36^ The index produced separate scores for fruit and vegetable consumption and took into account diversity and frequency of consumption of each included food. A later version of this index was used in the final study to investigate plant-based diversity using a dedicated indicator,^24^ and the index has since been validated against food and nutrient intakes from a country-specific FFQ.^57^

The remaining studies calculated diversity by assessing the number of unique plant-based food items or subgroups consumed, as reported in the assessments of plant-based food intake (ie, FFQ’s, 24-hour recall, food diaries, etc). There was heterogeneity in the reporting of plant-based food diversity in these articles (Table 2). Nineteen (44%) studies presented diversity as the total number of unique plant-based food items consumed.^16^^,^^19^^,^^20^^,^^22^^,^^26^^,^^28^^,^^32^^,^^37–39^^,^^44^^,^^46–50^^,^^53–55^ Diversity was defined as the total count of individual plant-based foods (eg, apple, pear, carrot, onion) consumed in a pre-defined time period unique to each study, ranging from 24 hours to 1 month (Table 2). One study did not provide sufficient details to determine the time frame for the diversity assessment.^49^ The majority of these studies did not consider frequency of consumption; consuming an item more than once in the prespecified time period contributed only once toward the score, for example, consuming an apple twice contributed just 1 point to the total score.

Fourteen (33%) studies expressed diversity as the total number of plant-based foods consumed within predefined subgroups, which were unique to each study.^5^^,^^17^^,^^21^^,^^23^^,^^25^^,^^27^^,^^29–31^^,^^35^^,^^40^^,^^45^^,^^51^^,^^52^ For example, subgroups might include leafy green vegetables, cruciferous vegetables, citrus fruits, etc, and plant-based diversity score calculations were based on the sum of the different types of plant-based foods consumed from each plant food group, (eg, fruits, vegetables, grains) within a defined time period unique to each study, ranging from 24 hours to 3 months (Table 2). Two studies assessing plant-based food diversity at the group level incorporated the Berry Index in their assessment of diversity.^17^^,^^31^ The Berry Index takes into account the evenness of distribution of foods within the diet. In these studies the Berry Index was calculated by counting the number of different plant-based food items consumed, divided by the number of items assessed, for each plant food subgroup, taking into account the distribution of foods across each subgroup, to yield an overall diversity score.^58^ Two studies presented diversity as the proportion of food items consumed within each predefined subgroup, whereby diversity scores represented the percentage of items consumed by participants within that subgroup.^21^^,^^27^^,^^40^

Four studies, all assessing diversity in multiple countries, utilized a variety of diversity indices that represented diversity of fruits and vegetables separately, at both the item and group level.^33^^,^^34^^,^^41^^,^^43^

Finally, 2 studies assessed plant-based diversity in a single question, the first assessing diversity of vegetables only (“How many different vegetables do you eat in a day?”)^15^ and the second assessing diversity of plants (“In an average week, how many different plants do you eat?”).^4^

Thirty-four (79%) studies had no minimum quantity cut-off that had to be met for a plant-based food item/group to be included in the plant-based diversity score (Table 3).^4^^,^^5^^,^^15^^,^^16^^,^^19–21^^,^^26–30^^,^^32–39^^,^^41^^,^^43–55^ Therefore, any quantity of consumption of a plant-based food item or group within the defined time frame counted toward diversity. In 2 studies it was unclear whether a minimum quantity had been used.^17^^,^^40^ There was heterogeneity in cut-offs used by studies that did specify a minimum quantity. Three studies defined the minimum quantity to be a recommended portion size that was specific to national guidelines in that country.^22^^,^^24^^,^^31^ Half a standard portion size was used as a cut-off in 1 study,^25^ and one-quarter of a standard portion size was used as a minimum quantity in another.^42^ One study required consumption of at least 15 g^23^ of a plant-based food item for it to be considered in the diversity score. Finally, 1 study specified a minimum consumption quantity of ≥0.1 cup equivalent for single items, or ≥0.2 cup equivalent for items in mixed dishes.^18^

**Table 3.** Reported Associations Between Health Outcomes, Determinants of Health, and Plant-Based Diversity

Study	Study design	Outcome(s) assessed	Plant-based food diversity assessment method (score range)	Classification of diversity score	Reported findings
Conklin et al (2014)^16^	Cross-sectional	Socioeconomic status	Sum of unique plant-based items consumed	Continuous score	Lowest fruit and vegetable diversity scores were significantly associated with lower education status, social class, and renting property (*P < *.001). Vegetable diversity differed between highest and lowest social classes by 2.9 vegetable types/month for males, and 2.5 vegetable types/month for females. Greater financial hardships were independently associated with lower diversity, particularly among women for fruits (*P < *.001) and among men for vegetables (*P < *.001)
Estaquio et al (2008)^40^	Prospective cohort	Demographic and lifestyle factors	Sum of unique plant-based items consumed	Proportion of foods consumed from each fruit/vegetable subgroup	Higher vegetable diversity was found in males and females with higher education level (*P = *.002), those cohabiting compared with living alone (*P < *.05), and in nonsmokers/previous male smokers compared with current smokers (*P < *.0001). Higher fruit diversity was found to be associated with higher education level for men (*P* * <* .001), but not women (*P = *.78). Higher fruit diversity was found for females cohabiting compared with those living alone (*P < *.001), smoking status in males and females (*P < *.0001), and physical activity (*P = *.03) and alcohol levels (*P < *.001) in males
Hoy et al (2020)^18^	Cross-sectional	Demographic and lifestyle factors	Sum of unique plant-based items consumed (0-no max)	Diversity terciles: None: 0; Low: 1-2; Moderate: 3-4; High: 5	Lowest fruit and vegetable diversity tercile had a higher proportion of 20-29-y-olds, people at <350% poverty income ratio, people with lower education, and more smokers, in comparison with highest fruit and vegetable diversity tercile. High fruit and vegetable diversity was associated with larger quantity of fruit and vegetable consumption
Leslie et al (2012)^22^	Cross-sectional	Breastfeeding and socioeconomic status	Sum of unique plant-based items consumed (fruit diversity: 0-20; vegetable diversity: 0-29)	Continuous	Higher fruit and vegetable diversity was observed in breastfeeding mothers, compared with non-breastfeeding mothers (*P < *.001). High socioeconomic position was associated with higher fruit diversity (β = 2.1, 95% CI 0.6-3.3, *P = *.05) and vegetable diversity (β = 8.7, 95% CI 6.6-10.9, *P = *.04), compared with low socioeconomic position
Baldwin et al (2021)^24^	Cross-sectional	BMI, healthcare costs, financial status	Fruit and Vegetable Variety Index (0-185)	Fruit diversity quintiles: Q1: 15.8 ± 6.5; Q2: 22.6 ± 6.4; Q3: 27.0 ± 6.0; Q4: 31.3 ± 6.0; Q5: 39.2 ± 7.1 Vegetable diversity quintiles: Q1: 42.3 ± 9.6; Q2: 54.8 ± 6.6; Q3: 61.5 ± 6.2; Q4: 67.7 ± 6.0; Q5: 77.9 ± 8.2 Total fruit and vegetable diversity quintiles: Q1: 58.1 ± 10.7; Q2: 77.4 ± 3.7; Q3: 88.6 ± 2.9; Q4: 99.1 ± 3.5; Q5: 117.0 ± 10.5	High fruit and vegetable diversity was significantly associated with fewer insurance-based health claims in women with normal BMI (4.3% [95% CI 1.9–6.8], and there were fewer health claims with every 10-point increase on FAVVA). The lowest total fruit and vegetable diversity quintile was significantly associated with increased likelihood of difficulty managing income, insurance-based health claims, and being overweight/obese compared with the highest total fruit and vegetable diversity quintile (*P < *.05)
Byrne et al (2018)^52^	Longitudinal cohort	Dietary quality	Sum of unique plant-based groups consumed (fruits 0-30; vegetables 0-36)	Continuous score: Fruit diversity score (0-30) Vegetable diversity score (0-36)	Higher diet quality was associated with higher fruit and vegetable diversity (*P < *.001)
Cano-Ibanez et al (2019)^25^	Cross-sectional	Nutrient intake adequacy	Sum of unique plant-based subgroups consumed (0-2 for each subgroup)	Quartiles. Further information NR	Higher risk of inadequate nutrient intake was shown for lowest fruit diversity quartile, compared with higher fruit diversity (OR 11.6, 95% CI 6.8-19.8), and for lowest vegetable diversity quartile compared with higher vegetable diversity (OR 14.0, 95% CI 10.6-18.7). No association was found for cereal diversity
Ellis et al (2018)^21^	Cross-sectional	Picky eating	Sum of unique plant-based items consumed	Proportion of foods consumed from each fruit/vegetable subgroup at least occasionally	Higher fruit diversity was significantly negatively associated with picky eating, compared with lower diversity (*r *= −0.32; *P < *.001). Higher vegetable diversity was significantly negatively associated with picky eating, compared with lower diversity (*r* = −0.38; *P < *.001)
Hazley et al (2022)^26^	Cross-sectional	Food neophobia	Sum of unique plant-based items consumed (0-no max)	Continuous score	Higher fruit and vegetable diversity was significantly associated with lower food neophobia scores (β −.05, 95% CI: −0.08 to −0.03, *P < *.001)
Jamison et al (2003)^55^	Case study	Dietary habits	Sum of unique plant-based items consumed (0-no max)	Continuous score	A total of 57% of participants ate ≥4 different types of fruits and vegetables daily. Higher fruit and vegetable intake was observed in females, compared with males (*P*-value NR)
Lopez Gonzalez et al (2021)^28^	Cross-sectional	Dietary intake and adequacy	Sum of unique plant-based items consumed (0- no max)	Overall diversity terciles: Low diversity: 11.5 ± 2.5; Moderate diversity: 16.0 ± 0.8; High diversity: 19.0 ± 1.0	Highest fruit and vegetable tercile was associated with lower odds of having inadequate intake of fiber (OR 0.1, 95% CI 0.1-1.2), and of 2 or more micronutrients (OR 0.2, 95% CI 0.1-0.2), compared with lowest diversity tercile. Highest fruit and vegetable tercile was associated with higher fiber, protein, carbohydrate, vitamin, mineral, and polyphenol intake, and lower total, monounsaturated, saturated fat, and trans fatty acids, compared with lowest diversity tercile. Highest fruit and vegetable tercile was associated with higher fruit, vegetable, legume, fish, and nut consumption, and lower cereal, olive oil, cookies, pastries, and alcohol consumption, compared with lowest diversity tercile
Salome et al (2020)^17^	Cross-sectional	Nutrient adequacy	(a) Sum of unique plant-based items/subgroups consumed, and taking into account (b) evenness of distribution (Berry Index)	Continuous score	Higher plant-based protein diversity (in terms of counts and evenness) was associated with higher nutrient adequacy scores (*r = *0.07 to 0.16, *P < *.05). Modeled substitutions showed highly diverse plant-based protein food consumption is required to maintain/improve nutrient adequacy when substituting animal protein with plant-based protein
Aljadani et al (2013)^36^	Longitudinal cohort	Body weight, nutrient intake	Fruit and Vegetable Index (0-333)	Fruit and vegetable frequency/diversity terciles: Low diversity: 34.6 ± 28.0; Moderate diversity: 83.1 ± 7.9; High diversity: 117.2 ± 18.9	High fruit and vegetable frequency/diversity led to significantly lower weight gain over 6 y than low frequency/diversity (β = −.72 (95% CI: −0.72 to −0.03; *P = *.041). Significant differences between terciles in total fat, saturated fat, protein, carbohydrate, and fiber intake (*P < *.05)
Bernstein et al (2002)^20^	Cross-sectional	Anthro-pometrics, nutrient intake, lipid profile	Sum of unique plant-based items consumed (0-no max)	Continuous score	Higher fruit and vegetable diversity was associated with higher BMI in women (β = 0.41, *P = *.01). Higher fruit and vegetable diversity was significantly positively associated with carbohydrate, fiber, vitamin C, vitamin B6, vitamin A, calcium, copper, potassium, and magnesium intake (*P < *.05). Higher fruit and vegetable diversity was associated with higher HDL cholesterol, and lower VLDL cholesterol and triglycerides in men (*P < *.05)
Oude Griep et al (2012)^39^	Prospective cohort	Coronary Heart Disease (CHD) risk, dietary intake	Sum of unique plant-based items consumed (fruits and vegetables: 0-22; fruits: 0-9; vegetables: 0-13)	[A] Fruit and vegetable diversity terciles: Low diversity: 8; Moderate: 9-12; High Diversity: 13. Fruit diversity terciles: Low: 3; Moderate: 4-6; High: 7 Vegetable diversity terciles: Low: 4; Moderate: 5-7; High: 8 [B] Continuous score	Fruit and vegetable diversity was not associated with CHD incidence (hazard ratio [HR] per 4 items 1.1, 95% CI; 0.9-1.3) or stroke incidence (HR per 4 items 0.9, 95% CI 0.8-1.1), after adjusting for age, gender, and lifestyle and dietary factors. Higher fruit and vegetable diversity was associated with higher vitamin C, carotenoids, flavonoids, and dietary fiber, compared with lower diversity. Fruit and vegetable intake was 2.5-fold higher in highest fruit and vegetable diversity tercile
Bhupathiraju et al (2013)^37^	Prospective cohort	CHD risk	Sum of unique plant-based items consumed (total: 0-30, Fruits: 0-11, Vegetables: 0-19)	Fruit and vegetable diversity quintiles: Women: Q1: 2.25; Q2: 3.38; Q3: 4.35; Q4: 5.49; Q5: 7.59 Men: Q1: 2.14; Q2: 3.29; Q3: 4.29; Q4: 5.52; Q5: 7.83	No association was found between quantity-adjusted fruit and vegetable diversity and CHD risk (relative risk [RR] for highest quintile 1.1, 95% CI 1.0-1.1, *P = *.34)
Conklin et al (2016)^5^	Prospective cohort	T2DM risk	Sum of unique plant-based subgroups consumed	Fruit: 0, 1, 2, 3 Vegetable: 0-1, 2, 3, 4 Grain: 0-1, 2	Higher fruit diversity led to 35% lower risk of T2DM, compared with lower fruit diversity (HR 0.7, 95% CI 0.5, 0.8). Higher vegetable diversity led to 33% lower risk of T2DM, compared with lower vegetable diversity (HR 0.7, 95% CI 0.5, 0.9). No association was found between grain diversity and T2DM risk
Cooper et al (2012)^38^	Prospective case cohort	T2DM risk	Sum of unique plant-based subgroups consumed (fruits and vegetables 0-117; fruits 0-58, vegetables 0-59)	Total fruit and vegetable diversity terciles: Low: 8.0 ± 1.8; Moderate diversity: 12.0 ± 0.8; High diversity: 16.3 ± 2.3 Fruit diversity terciles: Low: 2.0 ± 1.0; Moderate: 4.4 ± 0.5; High: 6.9 ± 1.2 Vegetable diversity terciles: Low: 5.5 ± 1.4; Moderate: 8.5 ± 0.5; High: 11.4 ± 1.5	Highest fruit and vegetable diversity tercile was associated with lower T2DM risk (HR 0.6, 95% CI 0.5-0.8). Highest fruit diversity tercile was significantly associated with lower T2DM risk, compared with lowest diversity tercile (HR 0.7, 95% CI 0.5-0.9, *P = *.002). Highest vegetable diversity tercile was significantly associated with lower T2DM risk (HR 0.8, 95% CI 0.6, 0.98, *P = *.03). Associations remained significant independent of known confounders and quantity intake
Buchner et al (2010)^33^	Prospective cohort	Lung cancer risk	Sum of unique plant-based items/groups consumed (total 0-40; Fruit items 0-14; vegetable items 0-26; Vegetable groups 0-8)	Total fruit and vegetable diversity quartiles: Q1: 0-10; Q2: 11-15; Q3: 16-22; Q4: 23-40 Fruit products diversity quartiles: Q1: 0-2; Q2: 3-5; Q3: 6-8; Q4: 9-14 Vegetable subgroups diversity quartiles (e.g., leafy): Q1: 0-4; Q2: 5-6; Q3: 7; Q4: 8 Vegetable items diversity quartiles: Q1: 0-6; Q2: 7-10; Q3: 11-15; Q4: 16-26	High vegetable subgroup diversity was associated with lower risk of lung cancer (HR, 0.8; 95% CI, 0.6-0.9, *P = *.02); an inverse association was shown for current smokers only (HR, 0.7; 95% CI, 0.6-0.9; *P = *.03). In current smokers, higher total fruit and vegetable diversity was associated with lower risk of squamous cell carcinomas (HR 0.9; 95% CI, 0.8-0.95)
Buchner et al (2011)^34^	Prospective cohort	Bladder cancer risk	Sum of unique plant-based items/groups consumed (total 0-40; fruit items 0-14; vegetable items 0-26; vegetable groups 0-8)	Total fruit and vegetable diversity terciles: Q1: 0-11; Q2: 12-19; Q3: 20-40 Fruit items diversity terciles: Q1: 0-3; Q2: 4-7; Q3: 8-14 Vegetable subgroup diversity terciles: Q1: 0-5; Q2: 6-7; Q3: 8 Vegetable items diversity terciles: Q1: 0-7; Q2: 8-13; Q3: 14-26	Highest fruit and vegetable diversity tercile was significantly associated with reduced risk of bladder cancer (HR = 1.3, 95% CI, 1.0-1.7, *P = *.05). No association was found when considering diversity as a continuous variable
Fernandez et al (1996)^47^	Case–control	Colorectal cancer risk	Sum of unique plant-based items consumed (0-no max)	Quantiles. Further Information NR	Higher vegetable diversity quantile was associated with lower colorectal cancer risk, compared with lowest quartile (RR 0.6, 95% CI 0.4-07, *P < *.01). No association was found between fruit diversity and colorectal cancer risk (*P = *.31)
Garavello et al (2008)^48^	Case–control	Oro-pharyngeal cancer risk	Sum of unique plant-based subgroups consumed (0-no max)	Fruit diversity terciles: Low: <4; Moderate: 4-5; High: ≥6 Vegetable diversity terciles: Low: <5; Moderate: 5-7; High: ≥8	High vegetable diversity was significantly associated with lower oropharyngeal cancer risk, compared with low diversity (OR 0.6; 95% CI 0.5–0.8). High fruit diversity was significantly associated with lower oropharyngeal cancer risk, compared with low diversity (OR 0.7; 95% CI 0.5–0.9)
Garavello et al (2009)^44^	Case–control	Laryngeal cancer risk	Sum of unique plant-based subgroups consumed (0-no max)	Fruit diversity quartiles: Q1: <3; Q2: 3 to <4; Q3: 4 to <6; Q4: ≥6 Vegetable diversity quartiles: Q1: <4; Q2: 4 to <6; Q3: 6-7; Q4: ≥7 Cereal diversity quartiles: Q1: <4; Q2: 4 to <6; Q3: 6-7; Q4: ≥7	Highest vegetable diversity quartile was significantly associated with lower laryngeal cancer risk, compared with lowest diversity quartile (OR = 0.4, 95% CI: 0.3–0.6). Highest fruit diversity quartile was significantly associated with lower laryngeal cancer risk, compared with lowest diversity quartile (OR = 0.4, 95% CI: 0.3–0.6). No association was found between cereal diversity and laryngeal cancer risk
Jansen et al (2004)^32^	Prospective cohort	Lung cancer risk	Sum of unique plant-based subgroups consumed (0-no max)	Fruit and vegetable diversity terciles: Low: 3-18; Moderate: 19-23; High: 24-33	Highest vegetable diversity tercile was significantly associated with a 36% lower cancer risk (RR 0.64, 95% CI 0.43-0.95, *P = *.02), and 49% lower non-lung epithelial cancer risk (RR 0.51; 95% CI 0.27–0.97). No association was found between fruit diversity and lung cancer risk
Jeurnick et al (2012)^43^	Prospective cohort	Gastric and esophageal cancer risk	Sum of unique plant-based items/subgroups consumed (0-no max)	Continuous	Higher fruit and vegetable diversity was associated with lower esophageal squamous cell carcinoma risk (HR per 2-product increment 0.88, 95% CI 0.79-0.97). Fruit and/or vegetable diversity was not associated with gastric cancer or esophageal adenocarcinomas risk
La Vecchia et al (1997)^45^	Case–control	Gastric cancer risk	Sum of unique plant-based items/subgroups consumed (0-no max).	Fruit diversity terciles: Q1: <2; Q2: 2; Q3: ≥3 Vegetable diversity quartiles: Q1: <5; Q2: 5; Q3: 6; Q4: ≥7	Highest fruit diversity tercile was significantly associated with lower gastric cancer risk (OR = 0.6; 95% CI 0.5–0.8, *P < *.001). Highest vegetable diversity quartile was significantly associated with lower gastric cancer risk (OR = 0.5; 95% CI 0.4–0.7, *P < *.001)
Leenders et al (2015)^41^	Prospective cohort	Colon and rectal cancer risk	Sum of unique plant-based items/subgroups consumed (0-no max)	Quartiles. Further information NR	Highest quartile of fruit and vegetable diversity (combined) was associated with decreased risk of colon cancer (HR = 0.97; 95% CI 0.92–1.02. *P = *.02). Highest fruit diversity quartile was significantly associated with higher rectal cancer risk, compared with lowest diversity quartile (HR 1.4, 95% CI 1.1-1.8, *P < *.01). No association was found between vegetable diversity and colon or rectal cancer risk
Lucenteforte et al (2008)^46^	Case–control	Oeso-phageal cancer risk	Sum of unique plant-based items consumed (0-no max)	Fruit diversity quartiles: Q1: <3; Q2: 3 to <4; Q3: 4 to <5; Q4: ≥5 Vegetable diversity quartiles: Q1: <4; Q2: 4 to <5; Q3: 5 to <7; Q4: ≥7 Cereal diversity quartiles: Q1: <4; Q2: 4 to <5; Q3: 5 to <7; Q4: ≥7	Higher fruit diversity was associated with lower squamous cell esophageal cancer risk (OR = 0.5, 95% CI 0.3-0.8, *P < .*001). Higher vegetable diversity was associated with lower squamous cell esophageal cancer risk, compared with lower diversity (OR = 0.3, 95% CI 0.2-0.6, *P < *.0001). No association was found between cereal diversity and esophageal cancer risk
McCann et al (1994)^50^	Case–control	Colon cancer risk	Sum of unique plant-based items consumed (0-no max)	Fruit diversity quartiles: Q1: ≤6; Q2: 7-8; Q3: 9-11; Q4: ≥12. Vegetable diversity quartiles: Q1: ≤10; Q2: 11-13; Q3: 14-16; Q4: ≥17 Grain diversity quartiles: Q1: ≤4; Q2: 5-6; Q3: 7-8; Q4: ≥9	No association was found between fruit, vegetable and grain diversity and colon cancer risk
Slattery et al (1997)^49^	Case–control	Colon cancer risk	Sum of unique plant-based items consumed (0-no max)	Fruit diversity quintiles: Women: Q1: <6; Q2: 6-8; Q3: 9-10; Q4: 11-14; Q5: > 14 Men: Q1: ≤5; Q2: 6-7; Q3: 8-9; Q4: 10-13; Q5: >13 Vegetables diversity quintiles: Women: Q1: <12; Q2: 12-17; Q3: 18-21; Q4: 22-26; Q5: > 26 Men: Q1: ≤11; Q2: 12-15; Q3: 16-19; Q4: 20-25; Q5: > 25 Wholegrains diversity quintiles: Women: Q1: <2; Q2: 2-3; Q3: 3.1-4; Q4: >4 Men: Q1: <2; Q2: 2-3; Q3: 3-4; Q4: >4 Refined grain diversity: Women: <6; Q2: 6-8; Q3: 8.1-9; Q4: 9.1-12; Q5: >12 Men: Q1: ≤5; Q2: 6-7; Q3: 8-9; Q4: 10-12; Q5: >12	Highest refined grain diversity quintile was associated with higher colon cancer risk in men (OR 1.7, 95% CI 1.3-2.3, *P < *.01), but not in women, compared with lowest diversity quintile Highest vegetable diversity quintile and wholegrain diversity quintile (separately) were associated with lower colon cancer risk, compared with the lowest diversity quintile in women only
Almeida de Souza et al (2018)^19^	Cross-sectional	Markers of inflammation	Sum of unique plant-based items consumed (0-no max)	Fruit diversity terciles: Low diversity: ≤9 categories/month; Moderate diversity: 10-11 categories/month; High diversity: ≥12 categories/month Vegetable diversity terciles: Low diversity: ≤6 categories/month; Moderate diversity: 7-12 categories/month; High diversity: ≥13 categories/month	High fruit diversity led to significantly higher IL-6, compared with low fruit diversity (OR 2.2, 95% CI 1.1-4.2, *P < *.05). High vegetable diversity led to significantly lower odds of having high CRP levels, compared with low vegetable diversity (OR 0.3, 95% CI 0.2–0.6, *P = *.004). No difference was found for C3 or overall inflammatory biomarker score in the fully adjusted models for fruit and vegetable diversity
De Castro-Mendez et al (2021)^23^	Cross-sectional	Autonomic nervous system activity	Sum of unique plant-based subgroups consumed (0-no max)	Continuous score	Higher vegetable diversity was significantly positively associated with average dilation velocity, a measure of sympathetic nervous system activity (β = .03, 95% CI 0.002-0.07). No association was found between fruit diversity and sympathetic nervous system activity
Fernandez et al (2016)^31^	Prospective cohort	Anthro-pometrics	Sum of unique plant-based items consumed, adjusted for evenness of food subgroup distribution (Berry Index) 0-30)	Continuous score	Higher fruit and vegetable diversity was not associated with annual increases in BMI (β = .011, *P = *.06)
Hurley et al (2010)^27^	Cross-sectional	Commercial baby food con-sumption effect on diversity	Sum of unique plant-based food items consumed (0-no max)	Continuous score	Higher fruit and vegetable diversity was found in infants aged 6-12 months who received commercial baby foods, compared with those who did not (*β* = .5, 95% CI 0.3-0.4, *P < *.001)
Marshall et al (2022)^30^	Cross-sectional	Socio-economic status, diet quality	Sum of unique plant-based subgroups consumed (fruits: 0-2, vegetables: 0-5; fruits and vegetables: 0-7)	Fruit and vegetable diversity terciles: Low: 0-1; Moderate: 2-3; High: ≥4. Fruit diversity groups: Low: 0; Moderate: 1; High: 2 Vegetable diversity terciles: Low: 0; Moderate: 1; High: 2	Higher fruit diversity was found in boys compared with girls (mean 1.12 vs 1.04, *P < *.05), and in higher socioeconomic status compared with lower socioeconomic status (*P < *.001). Higher fruit and vegetable diversity was associated with higher healthy eating scores (*β* = .8, SE = 0.2, *P < *.001)
Morison et al (2018)^54^	RCT	Baby-led weaning, food preferences	Sum of unique plant-based items consumed (0-no max)	Continuous score	A significantly higher fruit and vegetable diversity was found in infants following a baby-led weaning approach, compared with a control approach, at 24 months (difference in variety counts 2.0, 95% CI: 0.4-3.6, *P* value NR). No difference at 7 or 12 months
Perry et al (2015)^51^	Cross-sectional	Food neophobia	Sum of unique plant-based subgroups consumed (Fruit: 0-30, Vegetable: 0-36)	Continuous score	Lower fruit diversity and vegetable diversity was significantly associated with higher food neophobia scores (β= -0.16, *P = *.003; and β= -0.29, *P < *.001, respectively).
Ramsay et al (2017)^29^	Cross-sectional	Diet quality	Sum of unique plant-based subgroups consumed (0-7)	Continuous score based on subgroups: Fruit and vegetable diversity score (0-7)	Higher fruit and vegetable diversity was significantly associated with better dietary quality scores for total fruit, vegetable, and empty calories subscales (*P* value NR)
Rigal et al (2021)^53^	Nonrandomized controlled trial	Sensory education effect on fruit and vegetable diversity	Sum of unique plant-based items consumed	Continuous score	No effect of sensory education on fruit and vegetable diversity, compared with baseline
Skinner et al (2002)^42^	Prospective cohort	Early year food experiences effect on fruit and vegetable diversity	Variety index for Children (modified)	Continuous score	The number of vegetables liked by the mother was a significant predictor for vegetable diversity at 6-8 years old children (*R^2^*= 0.085, *P = *.0143). Breast-feeding duration, as well as early fruit diversity or fruit exposure, were a significant predictor for fruit diversity at 6-8 years old children
Venter et al (2020)^35^	Prospective cohort	Food allergy	Sum of unique plant-based subgroups consumed	Continuous score at 6 months and 9 months of age	Higher fruit and vegetable diversity at 6 and 9 months old was associated with lower odds of food allergy diagnosis over the first decade of life (OR 0.6, 95% CI 0.6-0.9, *P = *.0174 at 6 months; OR 0.8, 95% CI 0.7-0.9, *P = *.0163 at 9 months). No effect observed at 3 months old timepoint.
McDonald et al (2018)^4^	Cross-sectional	Gut microbiome	Self-reported number of different plants consumed. Single question 0-no max)	Low plant diversity group: Consume <10 plants/week High plant diversity group: Consume >30 plants/week	A significant positive association between molecular α-diversity and the high plant diversity group when compared with the low plant diversity group. High plant diversity was associated with sOTUs identified as putative SCFA producers. High plant diversity led to reduced abundance of antibiotic-resistance genes in comparison with low plant diversity
Radavelli-Bagatini et al (2022)^15^	Cross-sectional	Perceived stress levels	Self-reported daily vegetable diversity; single question (0-no max)	Continuous score	High correlation between vegetable diversity and total vegetable intake (rho = 0.8, *P = *.001). In multivariable-adjusted models for the impact of dietary factors on perceived stress, results indicated an association between high vegetable diversity and lower perceived stress that was largely related to the association resulting from total vegetable intake

Abbreviations: BMI, body mass index; CHD, coronary heart disease; CRP, C-reactive protein; FAVVA, Fruit and Vegetable Variety Index; HDL: high-density lipoprotein; RCT, randomized controlled trial; SCFA, short-chain fatty acid; sOTU, sub-Operational Taxonomic Unit; T2DM, type 2 diabetes mellitus; VLDL: very-low-density lipoprotein cholesterol.

### Outcomes Associated with Diversity of Plant-Based Food Consumption

Studies included in the review assessed a diverse range of health-related outcomes (Table 3). Regardless of whether studies defined and assessed plant-based food diversity at the item, subgroup, or group level, the majority of articles (38/43; 88%) reported associations between health outcomes and plant-based food diversity at the group level, for example, reporting an association between a specific health outcome and fruit or vegetable diversity. One article reported associations between health outcomes and plant-based protein diversity.^17^ Three articles reported associations between health outcomes and fruit and/or vegetable diversity, and in addition reported associations with vegetable subgroup diversity^33^^,^^34^ and grain subgroup diversity.^49^ One article reported diversity as overall plant-based diversity.^4^

#### Lifestyle Factors, Determinants of Health, and Plant-Based Food Diversity

Six (14%) studies investigated associations between plant-based diversity and socioeconomic, demographic, or lifestyle factors; of these 5 were cross-sectional studies,^16^^,^^18^^,^^22^^,^^24^^,^^30^^,^^40^ and 1 was a prospective cohort study.^40^ Higher fruit and vegetable diversity combined was associated with a reduction in insurance-based health claims made by women in the healthy body mass index (BMI) range.^24^ Lower education, lower social class, renting, and greater financial hardships were each independently associated with lower fruit or vegetable diversity in older adults in the United Kingdom, and this article also reported no association between these outcomes and fruit or vegetable quantity.^16^ Similarly, education level was positively associated with vegetable diversity in French adults.^40^ One article reported lower fruit and vegetable diversity was more common in younger adults (20–29 years) in comparison with older adults (≥60 years), black or other race/ethnic group in comparison with white, Hispanic, or Asian ethnic groups, smokers in comparison with nonsmokers, and in participant groups associated with several other socioeconomic risk factors, but the authors did not report whether these results were significant.^18^

#### Dietary Intake and Behavior and Plant-Based Food Diversity

Eleven (26%) studies investigated associations between plant-based diversity and dietary intake; 7 studies were cross-sectional,^17^^,^^20^^,^^25^^,^^28–30^ 2 were longitudinal cohort studies,^36^^,^^52^ 1 was a case study^55^ and 1 was a prospective cohort study.^39^ Four articles reported associations between plant-based diversity and nutrient intake.^20^^,^^28^^,^^36^^,^^39^ One article reported significantly higher intakes of protein, carbohydrates, and fiber, and lower intakes of total and saturated fat across tertiles of Fruit and Vegetable Index scores, with higher scores indicating higher diversity.^36^ Another reported that higher combined fruit and vegetable diversity was significantly positively associated with carbohydrate, fiber, vitamin C, vitamin B6, vitamin A, calcium, copper, potassium, and magnesium intake^20^; similarly, another cross-sectional study found that the highest combined fruit and vegetable tertile was associated with higher fiber, protein, carbohydrate, vitamin, mineral, and polyphenol intake, in addition to lower total fat, monounsaturated fat, saturated fat, and trans fatty acids, compared with the lowest diversity tertile.^28^ A prospective cohort study article reported higher combined fruit and vegetable diversity to be associated with higher vitamin C, carotenoids, flavonoids, and dietary fiber, compared with lower diversity.^39^

Three articles reported assessing the impact of plant-based diversity on diet quality, and all reported that higher plant-based diversity was associated with better dietary quality scores using a variety of tools.^29^^,^^30^^,^^52^

Three studies found associations between plant-based diversity and dietary adequacy. One study found an inverse relationship between the proportion of participants who had inadequate intake of ≥4 nutrients (according to North American dietary recommendations), and fruit or vegetable diversity scores separately.^25^ Similarly, 1 study found the highest combined fruit and vegetable tertile was associated with reduced risk of having inadequate intake of fiber, and of 2 or more micronutrients, compared with the lowest diversity tertile.^28^ The final study investigated the association between plant-based protein diversity and nutrient adequacy and found that higher diversity is significantly associated with higher nutrient adequacy scores.^17^

Three (7%) studies investigated relationships between plant-based diversity and eating behaviors in adults. Two of these studies were cross-sectional and investigated the impact of high levels of picky eating^21^ or food neophobia^26^ on fruit and vegetable diversity separately and combined, respectively. Both studies found significant inverse associations between these eating behaviors and fruit and vegetable diversity, whereby those who scored highly on either picky eating or food neophobia consumed significantly lower diversity of fruits and vegetables. Finally, a case study in chiropractic patients found insufficient consumption of diverse fruits and vegetables in this population but did not provide data to support this.^55^

#### Cardiometabolic Health and Plant-Based Food Diversity

Eight (19%) studies found outcomes relating to cardiometabolic health and their relationship to plant-based diversity. Five were prospective cohort studies,^5^^,^^31^^,^^37–39^ 2 were cross-sectional,^20^^,^^24^ and 1 was a longitudinal cohort study.^36^ Four of these studies investigated associations between plant-based diversity and anthropometric measures, 1 finding that higher combined fruit and vegetable diversity resulted in significantly lower weight gain over a follow-up period of 6 years, in comparison with low fruit and vegetable diversity,^36^ and another finding an inverse association between combined fruit and vegetable diversity quintiles and likelihood of having a BMI classified as overweight/obese.^24^ In contrast, another study found higher combined fruit and vegetable diversity to be associated with higher BMI in elderly women.^20^ Finally, a study in school-aged children found no association between combined fruit and vegetable diversity and BMI.^31^

Two studies found no association between combined fruit and vegetable diversity and risk of coronary heart disease.^37^^,^^39^ Two studies found that higher fruit or vegetable diversity separately were associated with reduced risk of type 2 diabetes, in comparison with low fruit and vegetable diversity,^5^ and in 1 of these studies the effect was also seen for combined fruit and vegetable diversity.^38^ One study found that higher combined fruit and vegetable diversity was associated with higher high-density lipoprotein (HDL) cholesterol and lower very low-density lipoprotein (VLDL) cholesterol and triglycerides in elderly men.^20^

#### Cancer Risk and Plant-Based Food Diversity

Twelve (28%) studies investigated associations between plant-based food diversity and cancer risk; these included 7 case–control studies^44–50^ and 5 prospective cohort studies.^32–34^^,^^41^^,^^43^ Higher diversity of plant-based foods was found to be significantly associated with a decreased risk of lung cancer in 2 studies (both vegetable diversity),^33^ colon cancer (fruit and vegetable diversity combined),^41^ and colorectal cancer (vegetable diversity),^47^ bladder cancer (fruit and vegetable diversity combined),^34^ oropharyngeal cancer (fruit or vegetable diversity, separately),^48^ laryngeal cancer (fruit or vegetable diversity, separately),^44^ and gastric cancer (fruit or vegetable diversity, separately).^45^ One study found higher combined fruit and vegetable diversity was associated with a lower risk of esophageal squamous cell carcinoma,^43^ while another study found an association between a reduced risk of the same form of cancer and higher fruit or vegetable diversity, but results were not reported for combined fruit and vegetable diversity.^46^

Three studies found positive associations between plant-based diversity and colon or rectal cancer risk; 2 found an association between higher grain^47^ or refined grain^49^ diversity and increased risk of colorectal or colon cancer, respectively; the third found higher diversity of fruit consumption to be associated with increased risk of rectal cancer.^41^

Various studies found no association between plant-based food diversity and laryngeal cancer risk (cereal diversity),^44^ lung cancer risk (fruit diversity),^32^ gastric cancer or esophageal adenocarcinoma (fruit and/or vegetable diversity),^43^ or esophageal cancer (cereal diversity).^46^ Four studies found no association between plant-based diversity and colon or rectal cancer risk, specifically vegetable diversity,^41^ fruit diversity,^47^ fruit, vegetable, and grain diversity (separately),^50^ and vegetable, fruit and wholegrain diversity (separately).^49^

#### Health Outcomes in Early Life and Adolescence, and Plant-Based Food Diversity

Nine (21%) studies included infants or children ≤12 years,^23^^,^^27^^,^^29^^,^^31^^,^^35^^,^^42^^,^^51^^,^^53^^,^^54^ and a further 2 studies included adolescents (12–18 years).^19^^,^^30^ These studies assessed a variety of outcomes relating to early life development, food behaviors, and risk of chronic disease. Six of these studies investigated relationships between plant-based diversity and early life exposures, and the development of eating behaviors. A cross-sectional study found that consumption of commercial baby food was associated with a higher consumption of combined fruit and vegetable diversity in infants aged 6–12 months, in comparison with infants who did not receive commercial baby food.^27^ An RCT found significantly higher combined fruit and vegetable diversity consumed by 24-month-old infants randomized to a baby-led weaning group, in comparison with a control feeding approach.^54^ Another cross-sectional study in children aged 24 months who scored highly for food neophobia found that this was associated with significantly lower fruit or vegetable diversity separately, in comparison with children without food neophobia.^51^ Another RCT in which it was hypothesized that a sensory education program may reduce food neophobia in children aged 7–11 and this would in turn increase combined fruit and vegetable diversity found that there was no impact of the intervention on this outcome.^53^ A prospective cohort study found that vegetables being liked by mothers and duration of breast feeding were significant predictors of vegetable diversity and fruit diversity, respectively, in children aged 6–8 years.^42^ Finally, higher combined fruit and vegetable diversity at 6 and 9 months of age was found to be associated with lower odds of food allergy diagnosis over the first decade of life.^35^

Two cross-sectional studies investigated the impact of plant-based diversity on risk factors for chronic disease, specifically markers of inflammation in adolescents^19^ and autonomic nervous system (ANS) activity in children.^23^ The former study found that higher vegetable diversity was associated with lower levels of C-reactive protein in adolescents.^19^ The latter study found that higher vegetable diversity was positively associated with average dilation velocity, a measure of the ANS response, so this may represent an important early life dietary factor for the regulation of health.^23^

#### Other Health-Related Outcomes and Plant-Based Food Diversity

Outcomes reported in only 1 article each included gut microbiota,^4^ and perceived stress.^15^ A cross-sectional study found that self-reported consumption of >30 plant-based foods per week was associated with increased gut microbial α-diversity (in comparison with the gut microbial α-diversity in those consuming ≤10 plant-based foods per week).^4^ Authors also identified several species of bacteria associated with the group consuming >30 plants per week, all of which functioned as short-chain fatty acid producers in the gut.^4^ Another study found significantly lower odds of having high perceived stress in those in the highest diversity quartile for several subgroups, in comparison with those in the lowest diversity quartile.^15^

## DISCUSSION

The current scoping review was conducted in light of the increasing interest in diversity of plant-based food intake and its perceived benefits for health. This is the first review of its kind and aimed to identify studies investigating the impact of plant-based food diversity on human health outcomes via a systematic search of the literature, and to characterize definitions of plant-based foods and plant-based food diversity, methods used for assessment of plant-based food consumption and plant-based food diversity, and outcomes investigated, including key findings. This scoping review highlights the urgent need for a consensus definition of what constitutes plant-based foods and plant-based food diversity, in addition to the need for validated measurement tools and standardization of reporting methods for assessment of plant-based food intake and diversity in research studies. Importantly, it also emphasizes the lack of high-quality evidence on the impact of diverse plant-based food consumption in health and disease.

The vast majority of studies considered only a limited selection of plant-based food groups when defining plant-based food diversity, namely fruits and vegetables only, with a small number of studies also including grains. This is likely a result of the emphasis of public health messaging on the importance of consuming a sufficient quantity of fruits and vegetables for health in recent decades.^59^^,^^60^ Plant-based food groups frequently omitted from definitions included legumes, herbs and spices, nuts and seeds, plant-based fats and oils (eg, olive oil), and plant-based beverages (eg, tea and coffee), all of which have well-documented benefits for human health that are often unique to a particular plant-based group. For example, a meta-analysis showed that fiber from wholegrains was associated with a reduction in colon cancer, yet fiber from fruit and vegetables didn’t share this same level of protection^61^; in contrast, another study showed that fiber from cereals and fruits provided greater protection from the development of diverticular disease than fiber from vegetables,^62^ suggesting that the health benefit associated with a food may depend on the specific form of a nutrient within that food, in this case the specific type of fiber. This reinforces the importance of exploring the biological benefits of a diverse intake of all plant-based foods to maximize health outcomes. In addition, studies differed in whether they consider plant-based food items (eg, apples, oranges, onions, carrots), plant-based food subgroups (eg, citrus fruits, cruciferous vegetables), or plant-based food groups (eg, fruits, vegetables, grains) in their definitions of diversity.

The assessment of diversity in terms of plant-based food subgroups or groups may appear advantageous, in that it allows identification of whether diversity within a specific plant food group may be responsible for a particular health effect, as reported for studies investigating intakes of specific plant-based foods and risk of disease,^61^^,^^62^ or whether diversity of all or any plant-based foods is favorable. Another advantage of assessing plant-based food diversity in terms of groups is that it facilitates assessment of evenness of distribution of foods contributing to diversity, across the groups, as was presented in 2 articles identified in the current review.^17^^,^^31^ Consideration of evenness ensures that diversity reflects contributions of foods from all plant-based food groups, in contrast to a potential scenario in which diversity could reflect a large number of plant-based foods consumed from a single group, for example fruits, and very few from the remaining groups of other healthful plant-based foods. A disadvantage of measurement of diversity at the food group or subgroup level is that, depending on the groups or subgroups included, there is a loss of information on the specific plant-based food items consumed. For example, studies have asked participants to indicate how many different citrus fruits, cruciferous vegetables, etc, they consumed per week, rather than asking participants about all the specific food items that are contained within these food subgroups, therefore losing valuable data on specific plant-based food items consumed. In addition, this may lead to misreporting of diversity, as it depends on participants having very good knowledge and recall ability for the plant-based food items included in each plant-based food group or subgroup. Plant-based food groups or subgroups defined by studies included in this review are often reflective of local diet, which limits the applicability of the findings to wider populations. The assessment of diversity at the food item level ensures detailed capture of all plant-based foods consumed within the diet, and therefore may be a more accurate measurement of plant-based food diversity. In addition, it would still facilitate subsequent analysis of the food items consumed at the subgroup or group level. However, this would require inclusion of an extensive list of plant-based food items, to allow for standardization of assessment and comparison of results across population groups and countries that consume vastly different diets, therefore increasing participant and analysis burden. To ensure optimal assessment of diversity, it is recommended that a combination of plant-based food items and subgroups are assessed.

Studies varied greatly in methods of measurement of plant-based food consumption and diversity. Tools used to measure plant-based food intake included FFQs, 24-hour recall, and food diaries, with the majority of studies stating the use of tools validated for assessment of dietary intake (Table 3). While these tools may indeed be validated against dietary biomarkers of energy intake, such as 24-hour urinary nitrogen intakes, there are currently no validated tools or assessment methods for measurement of plant-based food diversity.^63^ This is in part due to a lack of objective measures of food intake and diversity. The advantages and disadvantages of each method for assessment of dietary intake have been extensively discussed.^64^ In the context of this review, each method has further advantages and disadvantages to consider when it is used for the assessment of plant-based food intake and diversity. FFQs were favored by almost half of the studies; however, assessment of the diversity of plant-based foods using an FFQ is inherently limited by the number of food items included on the FFQ, and by the combining of foods within a single FFQ question (eg, apples and pears), which can result in inaccurate representation of plant-based food diversity. In the case of studies utilizing FFQs specific to the local diet, this further limits the generalizability of the findings and thus the impact of the research. The next most frequently used method was the 24-hour recall, which is inappropriate for the assessment of plant-based food diversity as it does not reflect the dynamic nature of diversity over time, whereby participants likely consume different foods on different days. Food diaries may be the most appropriate dietary assessment method for estimating plant-based food intake and diversity, as they can accurately and thoroughly capture plant-based food intake over a period of time when used correctly; however, food diaries are burdensome to participants and trial staff, and therefore they are not always feasible in a large trial setting or clinical practice. In addition, it is likely that foods that are not perceived by individuals as contributing to nutrient intake, for example herbs and spices, or tea and coffee, may be under-reported in this method. Only 3 studies utilized an index of diversity specifically developed to assess plant-based diversity (VIC, FAVI, FAVVA), while a further 2 studies used a common biodiversity index (Berry Index). These diversity indices were applied to dietary assessment tools (FFQ, etc) to quantify diversity. Tools were specific to the population of interest in each study, and while all articles reported validation of the tool against other subjective measures of dietary intake or biomarkers of specific nutrients, none were validated to specifically assess plant-based food diversity.

Importantly, the duration of the assessment of plant-based food diversity differed greatly between studies, ranging from 24 hours to 1 month in studies in adults, and up to 3 months in 1 study in children.^35^ Duration is a crucial consideration when assessing plant-based food diversity, as the nature of diversity reflects the count of each different plant-based food consumed over a given time period. Unless an individual consumes precisely the same diet each day, the plant-based diversity of their diet over a 24-hour period will almost certainly be vastly different to the diversity of their own diet (or indeed another individual’s diet) over a different time period (eg, 48 h, 7 days, etc). This is a significant disadvantage, as it limits comparisons of findings between studies that have used different time periods for dietary assessment. In general, diversity assessment over shorter time periods may not capture the day-to-day variation that is likely to occur in western diets.

A small number of studies set a quantity threshold above which consumed foods would be considered towards the diversity count,^18^^,^^22–25^^,^^42^ but the majority did not. This may have been due to several reasons. First, small amounts of micronutrients,^65^ and potentially nonnutrient bioactives such as (poly)phenols,^66^ are required for essential biological functions; therefore, it is plausible that even small amounts of certain plant-based foods that are rich in these components may provide health benefits, and thus it was considered they should be included when assessing diversity. Second, given that different plant-based foods are typically consumed in different quantities (eg, spices vs vegetables), it could be deemed methodologically burdensome to set quantity thresholds for each individual plant-based food. It may be sensible for quantity thresholds to be set at the level of the plant-based food group, rather than food item, whereby lower-quantity thresholds are set for food groups that are micronutrient or bioactive nonnutrient dense (eg, herbs and spices) than for other food groups (eg, plant-based food beverages).

While the majority of studies included in this review assessed plant-based food diversity at the item, or subgroup level, the findings were most often reported at the level of the plant-based food group (ie, association between a health outcome and vegetable diversity, fruit diversity, etc). In addition, populations were commonly grouped into categories of diversity, such as tertiles representing low, moderate, and high diversity of plant-based food intake.^28^^,^^30^^,^^39^^,^^45^ Due to the variation in definitions, methods of measurement, and reporting of plant-based diversity outlined above, it is challenging to compare levels of diversity, and subsequent associations with health outcomes, across studies. These studies highlight the need for standardization of definitions and assessment methods, so that international standards for characterizing “low” and “high” diversity of plant-based foods can be set and compared between studies in countries with similar dietary patterns.

The majority of studies were observational in design, with findings indicating that plant-based diversity may be an important target for the improvement of health in the general population, independent of quantity.^33^ Health outcomes most extensively investigated included markers of cardiometabolic health, cancer risk, and dietary intake and adequacy. Higher fruit and/or vegetable diversity was consistently associated with lower risk of type 2 diabetes.^5^^,^^38^ Higher vegetable diversity appears to be protective against certain types of cancer; however, associations between fruit diversity and cancer risk varied across studies.^32–34^^,^^41^^,^^43–50^ In addition, lower plant-based diversity was associated with determinants of poorer health in several studies, including lower education level and socioeconomic status.^16^^,^^18^^,^^22^^,^^40^ Lower plant-based diversity was consistently associated with lower dietary adequacy and lower intakes of beneficial nutrients such as fiber.^17^^,^^25^^,^^28^^,^^36^^,^^39^^,^^52^ Taken together, these findings indicate plant-based diversity may be a potential target for overall dietary improvement and beneficial health outcomes; however, this remains to be confirmed in a systematic review and meta-analysis, or RCT. Despite a recent increase in the promotion of plant-based diversity for its beneficial effects on the gut microbiota and gastrointestinal health, this review identified only 1 observational study investigating the impact of plant-based diversity on the gut microbiota. The findings indicated an association between higher plant-based food diversity and higher gut microbial α-diversity, which is a marker of gut health^4^; however, no clinical outcomes were assessed. This highlights the need for future RCTs investigating the impact of plant-based food diversity on gastrointestinal and cardiometabolic health outcomes, to provide a scientific rationale for public health messaging in this field of nutrition.

While most studies found associations between higher plant-based diversity and favorable health outcomes, some results are inconsistent, and the overall findings must be interpreted with caution, due to the observational nature of the studies, in addition to the aforementioned limitations in the comparability of studies. For example, while 1 study found an inverse association between vegetable diversity and colorectal cancer risk,^47^ another found no association between vegetable diversity and risk of colon or rectal cancer separately.^41^ The former study assessed plant-based diversity at the item and group levels, using a mixture of structured questionnaires and 7-day food diaries over a period of 2 weeks, while the latter assessed plant-based diversity at the item level, using a structured nonvalidated questionnaire over a duration of 1 week. A longer duration of dietary assessment when considering any aspect of dietary diversity is more likely to yield higher estimates of diversity, as individuals have more opportunities to consume a greater number of different foods; similarly, the assessment of diversity from a food diary may provide a higher estimate of plant-based diversity than from administering a questionnaire that is limited to a certain number of prespecified food items only. The considerable methodological differences in assessment methods of dietary intake and indicators of plant-based diversity between studies may have contributed to the inconsistent results reported in these articles.

Over half of the studies included in this review were conducted before publication of the Strengthening the Reporting of Observational Studies in Epidemiology—Nutritional Epidemiology (STROBE-nut) statement.^67^ Thus, while the weight of evidence indicates plant-based diversity is beneficial for human health, robust conclusions cannot be reached due to heterogeneity of reporting in this area of nutrition.

The current review has several strengths. It is the first scoping review to systematically identify studies investigating the consumption of diverse plant-based foods and the impact on human health, and to subsequently characterize definitions, measurement methods, and outcomes. It employed a broad search strategy with few restrictions and adhered to gold standard guidelines for reporting scoping reviews as defined in the PRISMA-Scr statement.^12^^,^^13^ There are also several limitations to this review. First, the review was restricted to studies in high-income countries to optimize the comparability of findings between studies in populations with similar food systems and dietary patterns. While not within the scope of this scoping review, we acknowledge that economic development and consequent food system changes over time may affect the diversity of plant food consumption; this remains to be established in future research. Second, we did not include a quality assessment of the included studies or statistical synthesis of the results (a meta-analysis), as both were outside the scope of the current review; these should be assessed in a consequent systematic review and meta-analysis of the literature.^12^

Considering the findings of the current review, the following recommendations are proposed:

1. The publication of a consensus definition of “plant-based food diversity” incorporating all foods from plant sources under the following groups: Fruits, vegetables, grains, legumes, herbs and spices, nuts and seeds, plant-based fats and oils (eg, olive oil), and plant-based beverages (eg, tea and coffee);
2. A standardized approach to assessment of plant-based diversity, incorporating assessment of plant-based food items and subgroups, and clear guidelines for duration of reporting, quantity threshold, and frequency of consumption;
3. Research to establish robust objective measures of food intake, to enable validation of dietary measurement tools related to plant-based food diversity;
4. A standardized approach to reporting of plant-based diversity. It may be preferable to report a daily itemized count of individual plant food items in the first instance, with additional reporting of evenness of distribution of plant-based foods across the recommended plant-based groups;
5. Assessment of intakes of diverse plant foods in international cohort studies in order to define standard levels of diversity (eg, high, moderate, and low diversity) that can be applied in the general population;
6. A systematic review and meta-analysis of the evidence for the role of diverse plant-based food intake in health, incorporating quality assessment of included studies and subgroup analysis by population group (healthy, clinical, age, etc);
7. Randomized controlled trials investigating the impact of diverse plant-based food intake on health outcomes are warranted, to establish and investigate health effects identified in observational trials and explore mechanisms by which diversity affects human health. Results of trials should be analyzed using appropriate statistical methods to model the impact of dietary pattern, and explore any synergistic causal effects between plant-based food diversity and health outcomes.

## CONCLUSION

In conclusion, we have identified 43 original studies that emphasize a lack of standardization in the definition of plant-based food diversity and assessment methodology for diversity. While the impact of plant-based food diversity has been investigated for several health outcomes, findings are limited by contradicting evidence and the limitations in study designs. These, in combination with the increased interest in plant-based food diversity among the general population, highlight the urgent need to improve assessment and reporting of plant-based food diversity. We provide recommendations for future research efforts, to strengthen the evidence base and our understanding of the impact of plant-based food diversity on health, and to inform public health strategies for the benefit of population health.

## Supplementary Material

nuaf040_Supplementary_Data
