# Supplementary material for: Diversity of Plant-Based Food Consumption: A Systematic Scoping Review on Measurement Tools and Associated Health Outcomes
Source: Nutr Rev. 2025 Apr 29;83(10):1985–2014. doi: 10.1093/nutrit/nuaf040 (PMC12422005; doi:10.1093/nutrit/nuaf040)
Supplement: nuaf040_Supplementary_Data [file nuaf040_supplementary_data.zip › Revised Supplementary Materials_clean.docx]

**Supplementary Materials**

**Supplementary table 1.** Search strategy.

| **Database** | **Strategy** |
| --- | --- |
| OVID Medline | 1. diet* varie* diet* divers* diet* quality diet* indicat* diet* divers indicat* diet* quality and diversity 2. OR diet* divers* OR diet* varie* OR diet* quality OR diet* indicat* OR diet* divers indicat* • Search key words as below: plant food* plant-based plant based number of plant* fruit* and vegetable* plant* consum* quantity of plant* micronutrient* diet* adequa microbio* 3. OR “plant-based” OR “plant based” OR “plant food* OR number of plant* OR fruit* and vegetable* OR plant* consum* OR quantity of plant* OR micronutrient* OR diet* adequa OR microbio* 4. AND to combine 2 and 4. |
| Web of Science | - 1. ((ALL=("diet* divers*" )) OR ALL=("diet* varie*")) OR ALL=("diet* quality" )) OR ALL=("diet* indicat*")) OR ALL=("diet* divers* indicat*")) OR ALL=(diet quality and diversity)   2. (((((ALL=(plant based)) OR ALL=(plant-based)) OR ALL=("quantity of plant*")) OR ALL=("number of plant*")) OR ALL=("fruit* and vegetable*")) OR ALL=("plant* consum*")) OR ALL=(micronutrient*)) OR ALL=("diet* adequa*")) OR ALL=(microb*)) OR ALL=(microbio*)) OR ALL=("plant food*")   3. AND to combine 1 and 2. |

| **Supplementary table 2.** Characteristics of studies.   \| **Study** \| **Study design** \| **Location** \| **Population characteristics** \| **Sample size**  **(% female)** \| **Age (y)** \| \| --- \| --- \| --- \| --- \| --- \| --- \| \| Aljadani et al (2013) \| Longitudinal cohort \| Australia \| Young women. No diabetes, heart disease or cancer. Not pregnant. \| 4,287 (100) \| Baseline: mean 28, SD 2  Follow-up: mean 34, SD 2 \| \| Almeida de Souza et al (2017) \| Cross-sectional \| Portugal \| Adolescents aged 12-18y. Without known disease. \| 412 (52) \| Median 15  (25^th^-75^th^ centile: 13-16) \| \| Baldwin et al (2021) \| Cross-sectional \| Australia \| Women, aged 50-55y or 62-67y. \| 8,833 (100) \| Mean 53, SD 1.5 \| \| Bernstein et al (2002) \| Cross-sectional \| USA \| Elderly in care facility, aged >70y. Mobile. No progressing/terminal/acute illness. No severe cognitive impairment, morbid obesity, fracture of lower extremities, recent myocardial infraction, or on weight-loss diet. \| 98 (63) \| Mean 87, SD 0.6 \| \| Bhupathiraju et al (2013) \| Prospective cohort \| USA \| Female registered nurses aged 30-55y. Male healthcare professionals, aged 40-75y. No major disease. \| 113,276 (63) \| NR \| \| Buchner et al (2010) \| Prospective cohort \| Denmark, France, Germany, Greece, Italy, Netherlands, Norway, Spain, Sweden, UK. \| Predominantly aged 25-70 y. \| 452,187 (71) \| Mean 51, SD 10 \| \| Buchner et al (2011) \| Prospective cohort \| Denmark, France, Germany, Greece, Italy, Netherlands, Norway, Spain, Sweden, UK. \| Predominantly aged 25-70 y. \| 452,185 (71) \| Mean 51, SD 10 \| \| Byrne et al (2018) \| Longitudinal cohort \| Australia \| Children, healthy at birth. \| 337 (54) \| Mean 24, SD 1 \| \| Cano-Ibanez et al (2019) \| Cross-sectional \| Spain \| Aged 55-75 y or 60-75 y. BMI 27-40 kg/m^2^. Metabolic syndrome. \| 6,587 (48) \| 85% of 55-70 y  15% of >70 y \| \| Conklin et al (2014) \| Cross-sectional \| UK \| Aged ≥50 y. \| 9,580 (55) \| Mean 62, SD NR \| \| Conklin et al (2016) \| Prospective cohort \| UK \| Aged 45-74 y. No diabetes. \| 23,238 (55) \| Low: mean 58, SD 9  Moderate: 58, 9  High: 59, 9 \| \| Cooper et al (2012) \| Prospective case cohort \| UK \| Aged 40-79 y. Cases diagnosed with T2DM. \| Total: 3,704 (NR)  T2DM: 653 (NR) \| NR for total sample \| \| De Castro-Mendez et al (2021) \| Cross-sectional \| Portugal \| Children, aged 7-12y. \| 513 (50) \| Mean 9, SD 1 \| \| Ellis et al (2018) \| Cross-sectional \| USA \| College students in psychology courses \| 1,219 (63) \| Mean 19, SD 2 \| \| Estaquio et al (2008) \| Prospective cohort \| France \| Aged 45-62 y. \| 4,282 (45) \| Females: mean 51, SD 5  Men: mean 52, SD 5 \| \| Fernandez et al (1996) \| Case-control \| Italy \| Cases: colon and rectal cancer admitted to hospital.  Controls: admitted to hospital for acute, non-neoplastic, non-digestive tract diseases. \| Cases: 1,326 (46)  Controls: 2,024 (41) \| Cases: Median 62, range 20-74.  Controls: Median 55, range 19-74 \| \| Fernandez et al (2016) \| Prospective cohort \| USA \| Children in preschool with low-income carer background. No significant medical problems. \| 340 (51) \| Mean 4, SD 1 \| \| Garavello et al (2008) \| Case-Control \| Italy \| Cases: admitted to hospital with confirmed oropharyngeal cancer. Diagnosis in past 1 y.  Controls: admitted to hospital for acute, non-neoplastic diseases. No risk factors for oropharyngeal cancer. \| Cases: 805 (18)  Controls: 2,081 (37) \| Cases: median 58, range 22-18  Controls: median 58, range 19-79 \| \| Garavello et al (2009) \| Case-control \| Italy & Switzerland \| Cases: admitted to hospital with confirmed pharyngeal cancer. Diagnosis in past 1 y.  Controls: admitted to hospital for acute, non-neoplastic, non-related to smoking and alcohol consumption diseases. \| Cases: 527 (9) Controls: 1,297 (19) \| Cases: median 61, range 30-79  Controls: median 61, range 31-79 \| \| Hazley et al (2022) \| Cross-sectional \| Ireland \| Adults aged 18-90y. \| 1,088 (51) \| 18-35y: n 441 (41%)  36-51y: n 358 (33%)  52-64y: n 179, 17%  ≥65y: n 110 (10%) \| \| Hoy et al (2020) \| Cross-sectional \| USA \| Adults ≥20y, non-institutionalised. \| 10,064 (52) \| NR \| \| Hurley et al (2010) \| Cross-sectional \| USA \| Children from birth to 12 months old, whose mother was ≥18y. \| 733 (48) \| NR \| \| Jamison et al (2003) \| Case study \| Australia \| People attending chiropractic clinics. Further information NR. \| 782 (65) \| <46y: n 319, 41%  ≥46y: n 339, 46% \| \| Jansen et al (2004) \| Prospective cohort \| Netherlands \| Men aged 65-84y. \| 730 (0) \| Low diversity: mean 72, SD 6; moderate: mean 71, SD 5; high: 71, SD 5 \| \| Jeurnick et al (2012) \| Prospective cohort \| Denmark, France, Germany, Greece, Italy, Netherlands, Norway, Spain, Sweden, UK. \| Mostly 35-70 y. \| 452,269 (71) \| Mean 51, SD 10 \| \| La Vecchia et al (1997) \| Case-control \| Italy \| Cases: admitted to hospital, confirmed stomach cancer.  Controls: admitted to hospital for acute, non-neoplastic, non-digestive tract conditions. \| Cases: 746 (39) Controls: 2,053 (41) \| Cases: median 61, range 19-74  Controls: median 55, range 19-74 \| \| Leenders et al (2015) \| Prospective cohort \| Denmark, France, Germany, Greece, Italy, Netherlands, Norway, Spain, Sweden, UK \| Participants of the European Prospective  Investigation. \| 442,961 (71) \| Median 51, 10^th^-90^th^ percentile 38-63 \| \| Leslie et al (2012) \| Cross-sectional \| Australia \| First-time mothers attending parent groups. \| 529 (100) \| NR \| \| Lopez Gonzalez et al (2021) \| Cross-sectional \| Spain \| Men aged 55-75y. Women aged 60-75y. BMI 27-40 kg/m^2^.  Metabolic syndrome. No chronic disease. \| 6,647 (48) \| Low diversity: mean 65, SD 5; moderate: mean 65, SD 5; high: mean 65, SD 5 \| \| Lucenteforte et al (2008) \| Case-control \| Italy \| Cases: admitted to hospitals with diagnosed squamous cell esophageal cancer within past year.  Controls: admitted to hospital with acute, non-neoplastic conditions, not related to smoking or alcohol. \| Cases: 304 (10)  Controls: 743 (20) \| Cases: median 60, range 39-77  Controls: 60, range 36-77 \| \| Marshall et al (2022) \| Cross-sectional \| USA \| Middle and high-school students. \| 9,056 (49) \| NR \| \| McCan et al (1994) \| Case-control \| USA \| Cases: colon cancer.  Controls: residents in the neighbourhood of the case. \| Cases: 428 (52) Controls: 428 (52) \| NR \| \| McDonald et al (2018) \| Cross-sectional \| Primarily USA, UK, Australia. \| Self-selected citizen-scientist population. Healthy adults aged 20-69y. BMI 18.5-30 kg/m^2^. No IBD, diabetes or antibiotic use in past year. \| 219 (NR) \| NR \| \| Morison et al (2018) \| RCT \| New Zealand \| Infants/toddlers from birth to 24 months old. Not premature or congenital abnormality affecting feeding/growth at birth. \| Intervention: 105 (62)  Control 101: (48) \| NR \| \| Oude Griep et al (2012) \| Prospective cohort \| Netherlands \| Men and women aged 20-65 years. \| 20,069 (55) \| Low diversity: mean 41, SD 11; moderate: mean 42, SD 11; high: mean 42, SD11 \| \| Perry et al (2015) \| Cross-sectional \| Australia \| Toddlers aged 24 months old. \| 358 (54) \| Mean 24.0, SD 0.7 months \| \| Radavelli-Bagatini et al (2022) \| Cross-sectional \| Australia \| Men and women aged ≥ 25 years. \| 8,640 (50) \| Mean 48, SD 15 \| \| Ramsay et al (2017) \| Cross-sectional \| USA \| Children aged 2-5 years. No chronic disease. \| 2,595 (48) \| 2 years old: n 885, 28%  3 years old: n 555, 23%  4 years old: n 619, 26%  5 years old: n 536, 23% \| \| Rigal et al (2021) \| Non-randomised controlled trial \| France \| Children aged 7-11 years. \| Intervention: 39 (65) Control: 24 (35) \| 7 years: 3%; 8 y: 22%; 9 y: 32%; 10 y: 22%; 11 y 21% \| \| Salome et al (2020) \| Cross-sectional \| France \| Adults aged 18-64y. Excluding dietary under-reporting. \| 1,341 (58) \| NR \| \| Skinner et al (2002) \| Prospective cohort \| USA \| Infants and toddlers aged 2-24 months. Data collection on diversity at 6-8 years. \| 70 (NR) \| NR \| \| Slattery et al (1997) \| Case-control \| USA \| Cases: diagnosed colon cancer, aged 30-79 years at diagnosis.  Controls: NR \| Cases: 1,993 (45) Controls: 2,410 (47) \| NR^†^ \| \| Venter et al (2020) \| Prospective cohort \| UK \| Children aged 1-10 years \| 1 year old: 900  2 y: 858, 3 y: 891, 10 y: 827 (NR) \| NR^†^ \|   NR: Not reported; NR†: not reported in suitable form; BMI, Body Mass Index; IBD, Inflammatory Bowel Disease; T2DM, Type 2 Diabetes Mellitus  **Supplementary table 3.** Further detail of specific plant-based foods reported, exclusions, dietary guidelines and seasonality considerations in included studies. | | | | | |
| --- | --- | --- | --- | --- | --- | --- | --- | --- | --- | --- | --- | --- | --- | --- | --- | --- | --- | --- | --- | --- | --- | --- | --- | --- | --- | --- | --- | --- | --- | --- | --- | --- | --- | --- | --- | --- | --- | --- | --- | --- | --- | --- | --- | --- | --- | --- | --- | --- | --- | --- | --- | --- | --- | --- | --- | --- | --- | --- | --- | --- | --- | --- | --- | --- | --- | --- | --- | --- | --- | --- | --- | --- | --- | --- | --- | --- | --- | --- | --- | --- | --- | --- | --- | --- | --- | --- | --- | --- | --- | --- | --- | --- | --- | --- | --- | --- | --- | --- | --- | --- | --- | --- | --- | --- | --- | --- | --- | --- | --- | --- | --- | --- | --- | --- | --- | --- | --- | --- | --- | --- | --- | --- | --- | --- | --- | --- | --- | --- | --- | --- | --- | --- | --- | --- | --- | --- | --- | --- | --- | --- | --- | --- | --- | --- | --- | --- | --- | --- | --- | --- | --- | --- | --- | --- | --- | --- | --- | --- | --- | --- | --- | --- | --- | --- | --- | --- | --- | --- | --- | --- | --- | --- | --- | --- | --- | --- | --- | --- | --- | --- | --- | --- | --- | --- | --- | --- | --- | --- | --- | --- | --- | --- | --- | --- | --- | --- | --- | --- | --- | --- | --- | --- | --- | --- | --- | --- | --- | --- | --- | --- | --- | --- | --- | --- | --- | --- | --- | --- | --- | --- | --- | --- | --- | --- | --- | --- | --- | --- | --- | --- | --- | --- | --- | --- | --- | --- | --- | --- | --- | --- | --- | --- | --- | --- | --- | --- | --- | --- | --- | --- | --- | --- | --- | --- | --- | --- | --- | --- | --- | --- | --- | --- | --- | --- | --- | --- | --- | --- | --- |
| **Study** | **Specific plant-based food items assessed** | **Plant-based foods excluded** | **Regional/ National dietary guidelines considered**  **Yes (Y)/ No (N)** | **Seasonal considerations Yes (Y)/**  **No (N)** | **Exclusively plant-foods assessed in diversity  Yes (Y)/ No (N).** |
| Aljadani et al (2013) | Vegetables: Potato, pumpkin, cucumber, cauliflower, spinach, green beans, cabbage/Brussel sprouts, peas, broccoli, carrots, zucchini/eggplant, squash, capsicum, mushroom, tomatoes, lettuce, celery, avocado, onion, leek,spring onion, soybeans, tofu, baked beans, other beans.  Fruits: canned/frozen, pear, apricot, apple, orange/mandarin, grapefruit, banana, peach/nectarine/plum, mango, pineapple, strawberries, melon. | Potatoes cooked in oil | Y | N | N |
| Almedia de Souza et al (2017) | Fruits: apple, pear, orange, tangerine, melon, watermelon, banana, kiwi, strawberries, cherries, peach/plum, persimmon, fig/medlar/apricot, grape, papay, mango.  Vegetables: white/savoy cabbage, Portuguese cabbage, kale, broccoli, cauliflower, Brussel sprouts, turnip, spinach, green beans, lettuce, watercress, onion, carrot turnip, tomato, pepper, cucumber, pea/broad beans. | Canned fruit, fruit juices,starchy vegetables (potatoes), dried pulses | N | Y | Y |
| Baldwin et al (2021) | Vegetables: Potato, pumpkin, cucumber, cauliflower, spinach, green beans, cabbage/Brussel sprouts, peas, broccoli, carrots, zucchini/eggplant, squash, capsicum, mushroom, tomatoes, lettuce, celery, avocado, onion/leek/spring onion, soybeans/tofu, baked beans, other beans.  Fruits: canned/frozen, pear, apricot, apple, orange/mandarin/grapefruit, banana, peach/nectarine/plum, mango, pineapple, strawberries, melon. | Potatoes cooked in oil | Y | N | Y |
| Bernstein et al (2002) | Further information not reported | Condiments | N | N | N |
| Bhupathiraju et al (2013) | Citrus fruit (orange, grapefruit). Green leafy vegetables (raw spinach, cooked spinach, iceberg or head lettuce, romaine or leaf lettuce, kale, mustard, chard greens) Cruciferous (broccoli, cabbage, cauliflower, brussels sprouts, kale). Beta-carotene rich: (raw carrots, cooked carrots, winter squash, yams, raw and cooked spinach, cantaloupe, dried apricots) Lutein rich: (raw spinach, cooked spinach, kale, mustard, or chard greens). Lycopene rich: (tomatoes, tomato sauce, tomato juice). Vitamin C rich (cantaloupe, orange, grapefruit, strawberries, broccoli, green peppers). | Potatoes, soy, legumes. | Y | N | Y |
| Buchner et al (2010) | Vegetables: borage, chard, endive, lettuce, spinach, thistle, artichoke, aubergine, cucumber, eggplant, pepper, pumpkin, tomato, beetroot, carrot, celery, parsnip, radish, salsify, turnip broccoli, brussels sprouts, cabbage, cauliflower, kale, mushrooms, champignon, truffle, peas, corn, garlic, onion, shallot, asparagus, bamboo, fennel, leek Legumes: beans, chickpeas, lentils.  Fruit: Ggapefruit, lemon, lime, orange, tangerine,  apple, apricot, banana, cherry, date, fig, grape, kiwi, melon, nectarine, peach, pear, pineapple, plum, raisin, strawberry,  Fruit juices and tomato sauce. | Nuts, seeds, olives, legumes, potatoes and tubers. | N | Y | Y |
| Buchner et al (2011) | Vegetables: Borage, chard, endive, lettuce, spinach, thistle, artichoke, aubergine, cucumber, eggplant, pepper, pumpkin, tomato, beetroot, carrot, celery, parsnip, radish, salsify, turnip, broccoli, brussels sprouts, cabbage, cauliflower, kale, mushrooms, champignon, truffle, peas, corn, garlic, onion, shallot, asparagus, bamboo, fennel, leek Legumes: beans, chickpeas, lentils Fruit: grapefruit, lemon, lime, orange, tangerine, apple, apricot, banana, cherry, date, fig, grape, kiwi, melon, nectarine, peach, pear, pineapple, plum, raisin, strawberry  Fruit juices and tomato sauce. | Nuts, seeds, olives, legumes, potatoes and tubers. | N | Y | Y |
| Byrne et al (2018) | Fruits: apple, pear, berries, orange, lemon/lime, mandarin, peach, apricot, cherry, nectarine, plum, banana, pineapple, tropical, fig, grape, kiwi, melon, watermelon, lychee/persimmon/pomegranate, passionfruit, rhubarb, not specified, fruit salad, mix of fruits, fruit dish, puree, dried fruits, sultanas, infant fruit product.  Vegetables: Potato, broccoli, cabbage, cauliflower, beetroot, carrot, ginger, root vegetable, sweet potato, asparagus, celery, lettuce, spinach, sprouts, peas, beans, tomato, pumpkin, zucchini/squash, avocado, capsicum, chilli, cucumber, eggplant, mushroom, seaweed, onion/leek, mixed vegetable dish, vegetable dish. | Cereals | Y | N | N |
| Cano-Ibanez et al (2019) | Vegetables: Green, tomatoes, yellow, and mushrooms. Cereals: potatoes and refined or whole grain cereals (bread, pasta, rice, and breakfast cereals).  Fruits: all fresh fruit products - citrus fruits, tropical fruits, and other seasonal fruits. | Sauces, condiments, fruit juices | Y | N | N |
| Conklin et al (2014) | Further information not reported | Further information not reported | N | N | Y |
| Conklin et al (2016) | Fruits: Vitamin A-rich, citrus and berry, other. Vegetables: Vitamin A-rich, dark green leafy, starchy tubers, other/  Grains: Whole grains, non-wholegrains. | Potatoes | Y |  | N |
| Cooper et al (2012) | Fruits: including tinned, dried.  Vegetables. | Potatoes, fruit juice | Y | N | Y |
| De Castro-Mendez et al (2021) | Pulses (beans, peas, and lentils), Dark green leafy vegetables Other vitamin A-rich fruits and vegetables Other vegetables Other fruits | Further information not reported | N | N | Y |
| Ellis et al (2018) | Further information not reported | Further information not reported | N | N | Y |
| Estaquio et al (2008) | Fruits: Apple, medlar, pear, clementine, grapefruit, lemon, mandarin orange, orange, grapes, blackberry, blackcurrant, gooseberry, huckleberry, raspberry, redcurrant, strawberry Apricot, cherry, nectarine, peach, plum, cantaloupe, watermelon, banana, Fig, kiwi fruit, lychee, mango, papaya, passion fruit, persimmon, pineapple, pomegranate Apple juice, grape juice, grapefruit juice, lemon juice, orange juice, pineapple juice  Vegetables: Chicory, corn salad, curly endive, dandelion, French endive, lettuce, mixed green salad, watercress Brussels sprout, cabbage, pickled cabbage, spinach, avocado, cherry tomato, cucumber, eggplant, pepper, pumpkin, tomato, tomato puree, tomato sauce, zucchini, beetroot, carrot, celeriac, radish, salsify, turnip, Green pea, green bean, mixture of diced vegetables, asparagus, cardoon, celery, fennel bulb, garlic, heart of palm, leek, onion, rhubarb, shallot, scallion Artichoke, broccoli, cauliflower, Mushrooms. Bean sprout, Carrot juice, tomato juice, mixed vegetable juices | Dried fruits, potatoes, legumes | Y | Y | Y |
| Fernandez et al (1996) | Vegetables: potatoes, cabbages and cruciferous, carrots, spinach, lettuce, tomatoes, pulses, peppers.  Fruits: apples, citrus, watermelon. | Further information not reported | N | N | N |
| Fernandez et al (2016) | Fruits, Vegetables, Grains, Fruits and Vegetables combined. | Further information not reported | Y | N | N |
| Garavello et al (2008) | Vegetables raw and cooked,  pulses, potatoes; fruits: citrus, other fresh and cooked fruits and unsweetened juices | Further information not reported | N | N | N |
| Garavello et al (2009) | Fruit, vegetables, cereals. | Further information not reported | Y | N | N |
| Hazley et al (2022) | Wholegrains: whole grain breads, pasta, rice, flour, cereals etc. Fruits: Oranges, grapefruit, clementine, lemon, mango, melon, passionfruit, peach, blackberries, plums, strawberries, cherries apples, pears, bananas, fruit juices  Vegetables: broccoli, spinach, lettuce, cucumber carrots, pumpkin, sweetcorn, tomatoes, canned/fresh corn, onion, mushroom, Potatoes, boiled, mashed (excluding chipped or processed). | Fruit juices and smoothies, herbs, spices, condiments, fried/processed potatoes. | Y | N | N |
| Hoy et al (2020) | Fruits, vegetables, mixed dishes. | Potatoes, condiments, Sauces | Y | N | Y |
| Hurley et al (2010) | Fruits: apples, bananas, berries, melon, citrus, grape, peach, pear. Vegetables: green, yellow, green beans, starchy, white potatoes | 100% Fruit juice, dry cereals | N | N | N |
| Jamison et al (2003) | Fruits & Vegetables. | Further information not reported | N | N | Y |
| Jansen et al (2004) | Fruits: strawberries, berries, grapes, peaches, cherries, prunes, and apricots.  Vegetables: Further information not reported | Potatoes | Y | Y | Y |
| Jeurnick et al (2012) | Vegetables: borage, chard, endive, lettuce, spinach, thistle, artichoke, aubergine, cucumber, eggplant, pepper, pumpkin, tomato, beetroot, carrot, celery, parsnip, radish, salsify, turnip Broccoli, Brussels sprouts, cabbage, cauliflower, kale, Mushrooms, champignon, truffle, peas, corn, garlic, onion, shallot, asparagus, bamboo, fennel, leek Legumes: beans, chickpeas, lentils Fruit: grapefruit, lemon, lime, orange, tangerine  Apple, apricot, banana, cherry, date, fig, grape, kiwi, melon, nectarine, peach, pear, pineapple, plum, raisin, strawberry Fruit juices and tomato sauce. | Nuts, seeds, olives, legumes, potatoes and tubers. | Y | Y | Y |
| La Vecchia et al (1997) | Vegetables: potatoes, cabbages/cruciferae, carrots, spinach, lettuce, tomatoes, pulses, and peppers.  Fruits: apples, citrus fruits, and watermelon | Further information not reported | N | N | N |
| Leenders et al (2015) | Fruits: apple, pear, apricot/peach/nectarine, banana, strawberry, grape, cherry, orange mandarin, grapefruit, prune, fig, melon, watermelon, kiwi, pineapple.  Vegetables: Endive, green salad, lettuce, spinach, Swiss chard, artichoke, avocado, green beans, tomato, courgette, cucumber, eggplant, sweet pepper, gherkin, beetroot, carrot, celeriac, broccoli, Brussel sprouts, cabbage, red cabbage, white cabbage, cauliflower, mushrooms, corn, peas, broad beans, onion, garlic, asparagus, celery, fennel, leek. | Nuts, seeds, olives | Y | Y | Y |
| Leslie et al (2012) | Fruits & Vegetables | Fruit juice, potatoes | N | N | N |
| Lopez Gonzalez et al (2021) | Fruits: oranges, bananas, apples, strawberries, cherries, melon, kiwis, grapes, peaches.  Vegetables: chards, cabbages, lettuce, tomatoes, asparagus, onion, garlic. | Fruit and vegetable juices, potatoes, mushrooms, dried fruit | Y | N | Y |
| Lucenteforte et al (2008) | Fruits & Vegetables | Further information not reported | Y | Y | N |
| Marshall et al (2022) | Vegetables: Starchy (potatoes, corn, peas), orange (carrots, squash, sweet potatoes), green (lettuce, spinach, green beans, broccoli), other (peppers, tomatoes, courgette, asparagus, cabbage, cucumbers, mushrooms, eggplant, celery, artichoke, beans/legumes (pinto, baked, kidney, refried) Fruits: any whole fruit frozen/canned/dried, fruit juice. | Fried potatoes | Y | N | Y |
| McCan et al (1994) | Vegetables: all vegetables raw or cooked Fruits: all fruits, raw or cooked. Grains: cereals, grains, pastas, rice, breads. | Fried potatoes, sweet baked grain products | N | N | N |
| McDondald et al (2018) | Plants, including composite dishes and food products | Further information not reported | N | N | Y |
| Morison et al (2018) | Fruits & Vegetables | Fried potatoes | Y | N | N |
| Oude Griep et al (2012) | Fruits & Vegetables | Fruit & vegetable juices, potatoes, legumes, sauces | Y | Y | Y |
| Perry et al (2015) | Fruits & Vegetables | Further information not reported | Y | N | N |
| Radavelli-Bagatini et al (2022) | Fruits: apples/pears, oranges and citrus, bananas.  Vegetables: cruciferous (cabbage, Brussel sprouts, cauliflower, broccoli), allium (onion, leek, garlic), yellow/orange/red (tomato, pepper, carrot, beetroot, pumpkin), leafy green (lettuce, celery, silver beet, spinach), legumes (peas, green beans, bean sprouts, alfalfa sprouts, baked beans, soy, tofu). | Fruit juice, tinned fruit, dried fruit, potatoes fried | Y | N | Y |
| Ramsay et al (2017) | Fruits: citrus, non-citrus, 100% fruit juice.  Vegetables: dark green, red/orange, starchy (included french fries) and other. | Further information not reported | N | N | Y |
| Rigal et al (2021) | Vegetables: artichoke, avocado, beetroot, broccoli, sprouts, caper, carrots, cauliflower, onion, celery, chestnut, corn, christophine, cucumber, djondjon, eggplant, endive, garlic, giraumon, green beans, cabbage, pepper, salads, leek, mushroom, olives, peas, pickles, pois pay, pumpkin, seaweed, shallots, soy, spinach, squash, tomato, turnip, watercress, courgette.  Fruits: Apple, banana, blackberry, breadfruit, carambola, cherry, plum, clementine, coconut, grapes, grapefruit, grenadine, guava, khaki, kiwi, lime, lychee, mango, melon, orange, papaya, passion fruit, peach, pear, pineapple, prune, quince, raspberry, red berries, redcurrant, strawberry, sugar cane, watermelon, lemon. | Further information not reported | N | N | Y |
| Salome et al (2020) | Plant sources of protein including  Grains: refined/wholegrain. Legumes, nuts & seeds, fruits, vegetables, potatoes, other vegetables. | Further information not reported | N | N | N |
| Skinner et al (2002) | Fruits & Vegetables: | Fruit Juice | Y | N | Y |
| Slattery et al (1997) | Fruits: fresh, canned, dried.  Vegetables: legumes, potatoes, and other. Wholegrains | Fruit & vegetable juice | N | N | N |
| Venter et al (2020) | Vitamin A rich fruits & vegetables, non citrus/citrus fruits, strawberries. Other fruits & vegetables | Potato, tomato | N | N | N |

**Supplementary table 4.** Advantages and disadvantages of the identified assessment methods of plant-based food diversity.

| **Method** | **Description** | **Advantages** | **Disadvantages** |
| --- | --- | --- | --- |
| Sum of unique plant-based food items, sub-groups or groups consumed ^1–36^ | Simple count of plant-based food items, subgroups or groups or some combination of the three. In most cases, consuming the same food item at least once scores 1; not consuming the food item scores 0. Assessment of diversity takes place over a defined time period. The number of items assessed is determined by the method of dietary assessment (i.e. food diary vs FFQ vs 24-hr recall). In some cases, it is reported as the proportion of food items consumed from within a predefined sub-group or group. | - Value represents actual diversity intake. - Easy to interpret. - Applicable to all dietary assessment methods - Able to capture all plant-based food items consumed (if food diary is used as measure of dietary assessment) | - High analysis burden - Limited to number of food items in chosen dietary assessment method (unless food diaries used) - Duration of assessment is dependent on dietary assessment tool used - Comparability may be affected by dietary assessment tool used across studies - Recall bias |
| Fruit and Vegetable Index (FAVI)^37^ | Index of fruit and vegetable diversity derived from an FFQ (DQESv2). Consists of the fruit sub-scale (13 items) and the vegetable sub-scale (24 items). Consumption scored using the full range of the FFQ Likert scale from zero to nine, with “never” scored as zero and “≥3 times per day” scored as nine. Maximum possible score is 117 for the fruit sub-scale and 216 for the vegetable sub-scale, giving a maximum total FAVI score of 333 points. A higher FAVI score indicates a greater diversity and frequency of usual fruit and vegetable consumption. | - Comparable across studies - Quick and easy to complete - Can be applied to large samples - Low analysis burden (relative to other methods) - Indicates diversity over a long duration (12 months) | - Limited to number of food items assessed in FFQ - Limited to fruits and vegetables only - Applicable to one dietary assessment method only (DQESv2) - Recall bias |
| Fruit and Vegetable Variety Index (FAVVA)^38^ | Index of fruit and vegetable diversity derived from an FFQ (AES FFQ). Consists of the fruit sub-scale (13 questions) and the vegetable sub-scale (24 questions). Consumption scored using the full range of the FFQ Likert scale for each question (frequency options vary per question). Maximum possible score is 68 for the fruit sub-scale and 122 for the vegetable sub-scale, giving a maximum total FAVVA score of 190 points. A higher FAVVA score indicates a greater diversity and frequency of usual fruit and vegetable consumption. | - Comparable across studies - Quick and easy to complete - Can be applied to large samples - Low analysis burden (relative to other methods) - Indicates diversity over a long duration (6 months) | - Limited to number of food items assessed in FFQ - Limited to fruits and vegetables only - Applicable to one dietary assessment method (AES FFQ) - Recall bias |
| Variety Index for Children^39^ | Index of dietary diversity, by food group (including separate groups for fruits and vegetables), most commonly derived from repeated 24-hour recalls, or a combination of food diaries and 24-hr recalls (minimum of 3 days). The index uses the amount of a food consumed, as well as the diversity of foods consumed, over a 3-day period, and subsequently compares intake with recommendations from the Food Guide Pyramid^40^. To ensure dietary variety in scoring, the amount of any fruit or vegetable cannot exceed 3 servings over any 3-day period (i.e. if 4 servings of broccoli are consumed, the maximum score for broccoli would be 3). The total number of servings consumed over a three day period are divided by the recommended number of servings and expressed as a percentage. The maximum score is 100, with a higher score indicating greater diversity and adherence to Food Guide Pyramid minimum serving recommendations. | - Applicable to several dietary assessment methods - Potential for assessment of all plant-based food items, if food diaries are used. - Able to capture all plant-based food items consumed (if food diary is used as measure of dietary assessment) | - Country-specific (USA) - Limited to fruits and vegetables only - Limited to number of food items in chosen dietary assessment method (unless food diaries used) - Duration of assessment is dependent on dietary assessment tool used - Specific to children - High analysis burden - Comparability affected by varying dietary assessment tool used across studies - Recall bias (if FFQ or 24-hour recall used) |
| Berry Index ^41,42^  (also referred to as Simpson's Index or Berry-Simpson Index) | Index of biodiversity that accounts for both number of different food items consumed and their distribution (evenness) within food sub-groups, groups or total diet. | - Applicable to all dietary assessment methods - Able to capture all plant-based food items consumed (if food diary is used as measure of dietary assessment) - Can accounts for evenness across food sub-groups, groups or total diet. | - High analysis burden - Limited to number of food items in chosen dietary assessment method (unless food diary used) - Duration of assessment is dependent on dietary assessment tool used - Comparability may be affected by dietary assessment tool used across studies - Recall bias (if FFQ or 24-hour recall used) |
| Single question ^43,44^ | A single question assessing diversity of plants, or plant-based food subgroups or groups e.g. 1) How many different types of plants do you consume per week? 2) How many different types of vegetables do you consume per week? | - Independent of dietary assessment method - Low analysis burden | - Recall bias - Relies on respondent’s understanding of the definition of a plant-based food. - No information on the specific plant-based foods consumed |

AES FFQ; Australian Eating Survey Food Frequency Questionnaire; DQESv2: Dietary Questionnaire for Epidemiological Studies Version 2; FFQ: Food Frequency Questionnaire

**References**

1. Leslie DA, Hesketh KD, Campbell KJ. Breastfeeding mothers consume more vegetables and a greater variety of fruits and vegetables than non-breastfeeding peers : the influence of socioeconomic position. Nutrition and Dietetics. 2012;69(2):84-90. doi:10.1111/J.1747-0080.2012.01584.X

2. Oude Griep LM, Verschuren WMM, Kromhout D, Ocké MC, Geleijnse JM. Variety in fruit and vegetable consumption and 10-year incidence of CHD and stroke. Public Health Nutr. 2012;15(12):2280-2286. doi:10.1017/S1368980012000912

3. Lucenteforte E, Garavello W, Bosetti C, et al. Diet diversity and the risk of squamous cell esophageal cancer. Int J Cancer. 2008;123(10):2397-2400. doi:10.1002/ijc.23761

4. McCann SE, Randall E, Marshall JR, Graham S, Zielezny M, Freudenheim JL. Diet diversity and risk of colon cancer in western new york. Nutr Cancer. 1994;21(2):133-141. doi:10.1080/01635589409514311

5. Almeida-de-Souza J, Santos R, Lopes L, et al. Associations between fruit and vegetable variety and low-grade inflammation in Portuguese adolescents from LabMed Physical Activity Study. Eur J Nutr. 2018;57(6):2055-2068. doi:10.1007/S00394-017-1479-Y

6. Bernstein MA, Tucker KL, Ryan ND, et al. Higher dietary variety is associated with better nutritional status in frail elderly people. J Am Diet Assoc. 2002;102(8):1096-1104. doi:10.1016/S0002-8223(02)90246-4

7. Bhupathiraju SN, Wedick NM, Pan A, et al. Quantity and variety in fruit and vegetable intake and risk of coronary heart disease1-3. American Journal of Clinical Nutrition. 2013;98(6):1514-1523. doi:10.3945/ajcn.113.066381

8. Conklin AI, Forouhi NG, Suhrcke M, Surtees P, Wareham NJ, Monsivais P. Variety more than quantity of fruit and vegetable intake varies by socioeconomic status and financial hardship. Findings from older adults in the EPIC cohort. Appetite. 2014;83:248-255. doi:10.1016/J.APPET.2014.08.038

9. Cooper AJ, Khaw KT, Sharp SJ, et al. A prospective study of the association between quantity and variety of fruit and vegetable intake and incident type 2 diabetes. Diabetes Care. 2012;35(6):1293-1300. doi:10.2337/dc11-2388

10. Fernández E, D’avanzo B, Negri E, Franceschi S, Vecchia C la. Diet diversity and the risk of colorectal cancer in northern Italy. Cancer Epidemiol Biomarkers Prev. Published online 1996.

11. Garavello W, Giordano L, Bosetti C, et al. Diet diversity and the risk of oral and pharyngeal cancer. Eur J Nutr. 2008;47(5):280-284. doi:10.1007/S00394-008-0722-Y

12. Garavello W, Lucenteforte E, Bosetti C, et al. Diet diversity and the risk of laryngeal cancer: a case-control study from Italy and Switzerland. Oral Oncol. 2009;45(1):85-89. doi:10.1016/J.ORALONCOLOGY.2008.02.011

13. Hazley D, McCarthy SN, Stack M, et al. Food neophobia and its relationship with dietary variety and quality in Irish adults: Findings from a national cross-sectional study. Appetite. 2022;169. doi:10.1016/J.APPET.2021.105859

14. Jamison JR. Dietary diversity: A case study of fruit and vegetable consumption by chiropractic patients. J Manipulative Physiol Ther. 2003;26(6):383-389. doi:10.1016/S0161-4754(03)00071-X

15. Jansen MCJF, Bas Bueno-De-Mesquita H, Feskens EJM, Streppel MT, Kok FJ, Kromhout D. Quantity and variety of fruit and vegetable consumption and cancer risk. Nutr Cancer. 2004;48(2):142-148. doi:10.1207/s15327914nc4802_3

16. López-González L, Becerra-Tomás N, Babio N, et al. Variety in fruits and vegetables, diet quality and lifestyle in an older adult mediterranean population. Clin Nutr. 2021;40(4):1510-1518. doi:10.1016/J.CLNU.2021.02.024

17. Morison BJ, Heath ALM, Haszard JJ, et al. Impact of a Modified Version of Baby-Led Weaning on Dietary Variety and Food Preferences in Infants. Nutrients. 2018;10(8). doi:10.3390/NU10081092

18. Rigal N, Salmon-Legagneur A, Hébel P, Cassuto D, Politzer N. Effects of a family-based sensory education on vegetable and fruit variety in children. Food Qual Prefer. 2021;93:104258. doi:10.1016/J.FOODQUAL.2021.104258

19. Slattery ML, Berry TD, Potter J, Caan B. Diet diversity, diet composition, and risk of colon cancer (United States). Cancer Causes and Control. 1997;8(6):872-882. doi:10.1023/A:1018416412906

20. Byrne R, Yeo MEJ, Mallan K, Magarey A, Daniels L. Is higher formula intake and limited dietary diversity in Australian children at 14 months of age associated with dietary quality at 24 months? Appetite. 2018;120:240-245. doi:10.1016/J.APPET.2017.09.002

21. La Vecchia C, Muñoz SE, Braga C, Fernandez E, Decarli A. Diet diversity and gastric cancer. Int J Cancer. 1997;72(2):255-257. doi:10.1002/(SICI)1097-0215(19970717)72:2<255::AID-IJC9>3.0.CO;2-Q

22. Cano-Ibáñez N, Gea A, Martínez-González MA, et al. Dietary Diversity and Nutritional Adequacy among an Older Spanish Population with Metabolic Syndrome in the PREDIMED-Plus Study: A Cross-Sectional Analysis. Nutrients. 2019;11(5). doi:10.3390/NU11050958

23. Conklin AI, Monsivais P, Khaw KT, Wareham NJ, Forouhi NG. Dietary Diversity, Diet Cost, and Incidence of Type 2 Diabetes in the United Kingdom: A Prospective Cohort Study. PLoS Med. 2016;13(7). doi:10.1371/journal.pmed.1002085

24. Mendes F de C, Paciência I, Rufo JC, et al. Increasing Vegetable Diversity Consumption Impacts the Sympathetic Nervous System Activity in School-Aged Children. Nutrients. 2021;13(5). doi:10.3390/NU13051456

25. Marshall AN, Ranjit N, Van Den Berg A, Gill M, Hoelscher DM. Associations between variety of fruits and vegetables consumed, diet quality, and sociodemographic factors among 8th and 11th grade adolescents in Texas. Public Health Nutr. 2022;26(2):351-362. doi:10.1017/S1368980022001690

26. Perry RA, Mallan KM, Koo J, Mauch CE, Daniels LA, Magarey AM. Food neophobia and its association with diet quality and weight in children aged 24 months: A cross sectional study. International Journal of Behavioral Nutrition and Physical Activity. 2015;12(1). doi:10.1186/s12966-015-0184-6

27. Ramsay SA, Shriver LH, Taylor CA. Variety of fruit and vegetables is related to preschoolers’ overall diet quality. Prev Med Rep. 2017;5:112-117. doi:10.1016/j.pmedr.2016.12.003

28. Venter C, Maslin K, Holloway JW, et al. Different Measures of Diet Diversity During Infancy and the Association with Childhood Food Allergy in a UK Birth Cohort Study. J Allergy Clin Immunol Pract. 2020;8(6):2017-2026. doi:10.1016/J.JAIP.2020.01.029

29. Ellis JM, Galloway AT, Zickgraf HF, Whited MC. Picky eating and fruit and vegetable consumption in college students. Eat Behav. 2018;30:5-8. doi:10.1016/J.EATBEH.2018.05.001

30. Estaquio C, Druesne-Pecollo N, Latino-Martel P, Dauchet L, Hercberg S, Bertrais S. Socioeconomic Differences in Fruit and Vegetable Consumption among Middle-Aged French Adults: Adherence to the 5 A Day Recommendation. J Am Diet Assoc. 2008;108(12):2021-2030. doi:10.1016/j.jada.2008.09.011

31. Hurley KM, Black MM. Commercial baby food consumption and dietary variety in a statewide sample of infants receiving benefits from the special supplemental nutrition program for women, infants, and children. J Am Diet Assoc. 2010;110(10):1537-1541. doi:10.1016/J.JADA.2010.07.002

32. Büchner FL, Bueno-de-Mesquita HB, Ros MM, et al. Variety in fruit and vegetable consumption and the risk of lung cancer in the European prospective investigation into cancer and nutrition. Cancer Epidemiol Biomarkers Prev. 2010;19(9):2278-2286. doi:10.1158/1055-9965.EPI-10-0489

33. Büchner FL, Bueno-De-Mesquita HB, Ros MM, et al. Variety in vegetable and fruit consumption and risk of bladder cancer in the European Prospective Investigation into Cancer and Nutrition. Int J Cancer. 2011;128(12):2971-2979. doi:10.1002/IJC.25636

34. Jeurnink SM, Büchner FL, Bueno-De-Mesquita HB, et al. Variety in vegetable and fruit consumption and the risk of gastric and esophageal cancer in the European Prospective Investigation into Cancer and Nutrition. Int J Cancer. 2012;131(6). doi:10.1002/IJC.27517

35. Leenders M, Siersema PD, Overvad K, et al. Subtypes of fruit and vegetables, variety in consumption and risk of colon and rectal cancer in the European Prospective Investigation into Cancer and Nutrition. Int J Cancer. 2015;137(11):2705-2714. doi:10.1002/IJC.29640

36. Katherine Hoy M, Clemens JC, Martin CL, Moshfegh AJ. Fruit and Vegetable Consumption of US Adults by Level of Variety, What We Eat in America, NHANES 2013-2016. Curr Dev Nutr. 2020;4(3). doi:10.1093/CDN/NZAA014

37. Aljadani HM, Patterson A, Sibbritt D, Hutchesson MJ, Jensen ME, Collins CE. Diet quality, measured by fruit and vegetable intake, predicts weight change in young women. J Obes. 2013;2013. doi:10.1155/2013/525161

38. Baldwin JN, Ashton LM, Forder PM, et al. Increasing Fruit and Vegetable Variety over Time Is Associated with Lower 15-Year Healthcare Costs: Results from the Australian Longitudinal Study on Women’s Health. Nutrients. 2021;13(8). doi:10.3390/NU13082829

39. Skinner JD, Carruth BR, Bounds W, Ziegler P, Reidy K. Do food-related experiences in the first 2 years of life predict dietary variety in school-aged children? J Nutr Educ Behav. 2002;34(6):310-315. doi:10.1016/S1499-4046(06)60113-9

40. Marcus JB. Nutrition Basics: What Is Inside Food, How It Functions and Healthy Guidelines. Culinary Nutrition. Published online 2013:1-50. doi:10.1016/B978-0-12-391882-6.00001-7

41. Fernandez C, Kasper NM, Miller AL, Lumeng JC, Peterson KE. Association of Dietary Variety and Diversity With Body Mass Index in US Preschool Children. Pediatrics. 2016;137(3). doi:10.1542/PEDS.2015-2307

42. Salomé M, De Gavelle E, Dufour A, et al. Plant-Protein Diversity Is Critical to Ensuring the Nutritional Adequacy of Diets When Replacing Animal With Plant Protein: Observed and Modeled Diets of French Adults (INCA3). J Nutr. 2020;150(3):536-545. doi:10.1093/JN/NXZ252

43. McDonald D, Hyde E, Debelius JW, et al. American Gut: an Open Platform for Citizen Science Microbiome Research. mSystems. 2018;3(3). doi:10.1128/mSystems.00031-18

44. Radavelli-Bagatini S, Sim M, Blekkenhorst LC, et al. Associations of specific types of fruit and vegetables with perceived stress in adults: the AusDiab study. Eur J Nutr. 2022;61(6):2929-2938. doi:10.1007/S00394-022-02848-5
